# “Shake ‘n Bake” Route to Functionalized Zr-UiO-66 Metal–Organic Frameworks

**DOI:** 10.1021/acs.inorgchem.1c01839

**Published:** 2021-09-02

**Authors:** Roberto D’Amato, Roberto Bondi, Intissar Moghdad, Fabio Marmottini, Matthew J. McPherson, Houcine Naïli, Marco Taddei, Ferdinando Costantino

**Affiliations:** † Dipartimento di Chimica Biologia e Biotecnologia, University of Perugia, Via Elce di Sotto 8, 06123 Perugia, Italy; ‡ International Iberian Nanotechnology Laboratory, Avenida Mestre José Veiga s/n, 4715-330 Braga, Portugal; § Laboratory of Advanced Materials, National Engineering School, Sfax University, P.B. 1173, 3038 Sfax, Tunisia; ∥ Energy Safety Research Institute, Swansea University, Fabian Way, SA1 8EN Swansea, U.K.; ⊥ Laboratory Physico Chemistry of the Solid State, Department of Chemistry, Faculty of Sciences of Sfax, Sfax University, P.B. 1171, 3000 Sfax, Tunisia; # Dipartimento di Chimica e Chimica Industriale, Università di Pisa, Via Giuseppe Moruzzi, 13, 56124 Pisa, Italy

## Abstract

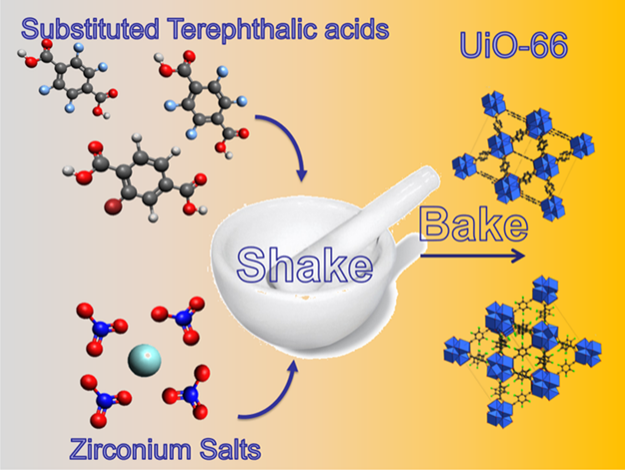

We report a novel synthetic procedure for the high-yield synthesis of metal–organic frameworks (MOFs) with **fcu** topology with a UiO-66-like structure starting from a range of commercial Zr^IV^ precursors and various substituted dicarboxylic linkers. The syntheses are carried out by grinding in a ball mill the starting reagents, namely, Zr salts and the dicarboxylic linkers, in the presence of a small amount of acetic acid and water (1 mL total volume for 1 mmol of each reagent), followed by incubation at either room temperature or 120 °C. Such a simple “shake ‘n bake” procedure, inspired by the solid-state reaction of inorganic materials, such as oxides, avoids the use of large amounts of solvents generally used for the syntheses of Zr-MOF. Acidity of the linkers and the amount of water are found to be crucial factors in affording materials of quality comparable to that of products obtained under solvo- or hydrothermal conditions.

## Abstract 2

Functionalized Zr-UiO-66 MOFs can be obtained with a new easy and quick procedure starting from a physical mixture of reagents and a little amount of liquid modulators.

## Introduction

The development of green and scalable procedures for the synthesis of metal–organic frameworks (MOFs) is currently considered the main factor to enable widespread industrial application and commercialization of these materials.^1,2^ The focus is primarily on the production of highly stable MOFs at low cost, in high yield, and fulfilling most of the requirements of sustainability and atom economy.^3,4^ Zirconium-based MOFs (Zr-MOFs) are currently considered benchmark materials for their high chemical and thermal stability, structural versatility, and employment in a vast range of applications, ranging from gas separation,^5−7^ catalysis,^8,9^ water sorption,^10,11^ proton conductivity,^12^ and drug delivery.^13^ Their structure is based on the different connectivities of hexanuclear clusters of the formula Zr_6_O_4_(OH)_4_^12+^ with polytopic carboxylic linkers, designing MOFs with variable degrees of connectivity and topologies, such as **fcu** (UiO-66 and MOF-801), **csq** (NU-1000), **reo** (DUT-67), and **spn** (MOF-808).^14−17^ Other topologies based on different secondary building units (SBUs), such as dodecanuclear clusters, were also recently reported.^18^ Zr-MOFs are often prepared employing hazardous solvents with high boiling points such as *N*,*N*-dimethylformamide (DMF), strong acids, and soluble chloride or nitrate metal salts.^19^ A remarkable effort has been recently made for ensuring safer and cleaner procedures for the synthesis of MOFs using different approaches able to minimize the use of hazardous reagents and solvents with high boiling points and the generation of large amounts of waste byproducts.^20−25^

Mechanochemistry is a well-established approach for performing clean and fast syntheses of a wide range of compounds, including metal–organic materials, avoiding common solvothermal routes and maximizing the atom economy.^26^ In particular, liquid-assisted grinding (LAG) or ionic-LAG is an efficient procedure that makes use of a small amount of solvents and/or metal-oxide precursors to enhance the crystallization kinetics.^27,28^ Accelerated aging is another solvent-free route which takes advantage of the relatively high vapor pressure of small amounts of organic solvents.^29,30^ Mechanochemical routes have recently been developed for the synthesis of many Zr-MOFs.^31^ In particular, the use of templating agents, water-based LAG, and extrusion resulted in the synthesis of Zr-MOFs of different topologies with high yield and high purity.^32^ However, in order to attain the desired phase, preformed Zr_6_O_4_(OH)_4_^12+^ or Zr_12_O_8_(OH)_8_^24+^ clusters already assembled with monocarboxylic ligands, such as acetate or methacrylate, are normally used.^32−34^ These clusters are often prepared using wet chemistry routes, adding a preliminary synthetic step to the procedure. Huang *et al.*(31) recently reported the ultrarapid (3 min) water-based LAG synthesis of nanocrystalline perfluorinated UiO-66 starting from a preformed methacrylate-based hexanuclear cluster and tetrafluoroterephthalic acid (F_4_-BDC). The authors found that other linkers such as terephthalic acid (BDC), 2-aminoterephthalic acid (NH_2_-BDC), and 2-bromoterephthalic acid (Br-BDC) failed to afford a crystalline product, attributing the higher reactivity of F_4_-BDC to its higher acidity, which enhances its solubility in water. Indeed, F_4_-BDC has recently been employed for the synthesis of UiO-66-type MOFs in water, even at room temperature (RT).^35−38^ Notably, Ye *et al.*(39) recently reported on a simple method to produce UiO-66 in high yield by grinding ZrOCl_2_·8H_2_O and BDC and subsequently heating the resulting mixture at 130 °C for 12 h. Attempts using ZrCl_4_ and Zr(NO_3_)_4_·5H_2_O as precursors failed to afford a crystalline product.

Inspired by these studies, we set out to investigate the synthesis of a range of functionalized UiO-66 analogues using a simple “shake ‘n bake” procedure, expression already used for the solid-state synthesis of mixed oxides performed by shaking or mixing the reagents with a low mechanical energy input and annealing them into a furnace for a prolonged time.^40^ An initial screening of different commercially available salts, namely, Zr(NO_3_)_4_·5H_2_O, ZrOCl_2_·8H_2_O, ZrO(NO_3_)_2_·4H_2_O, and ZrCl_4_, was first carried out. Further investigation on the influence of the AcOH/H_2_O ratio on the crystallinity of the compounds was also carried out. This screening was performed on the compounds prepared with Zr(NO_3_)_4_·5H_2_O as zirconium source, which afforded materials with high yield and crystallinity. Acidity of the linkers was found to be responsible for the different reactivities and quality of the obtained MOFs.

## Experimental Section

### Chemicals

**Figure 1:**
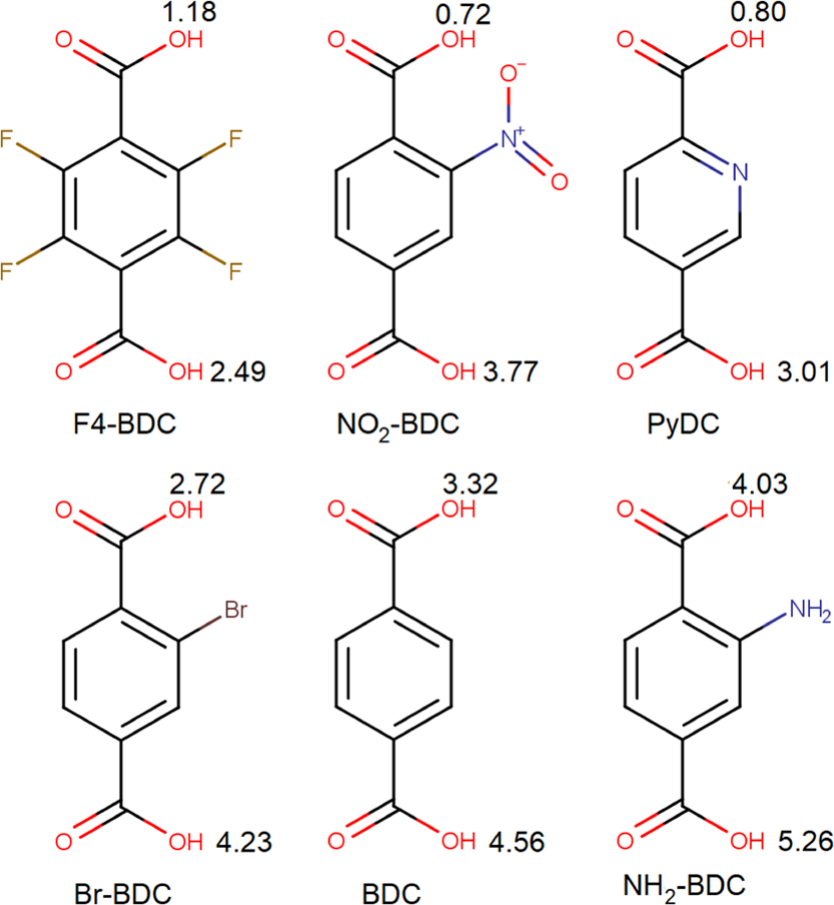
Molecular structure of the linkers used in this work. p*K*_a_ values are also included next to the corresponding carboxylic groups. The values were calculated using the online tool Chemicalize (chemicalize.com).

Zirconium nitrate pentahydrate [Zr(NO_3_)_4_·5H_2_O] and zirconium oxynitrate tetrahydrate [ZrO(NO_3_)_2_·4H_2_O] were supplied by Carlo Erba. Zirconium oxychloride octahydrate (ZrOCl_2_·8H_2_O) and 2-nitroterephthalic acid were supplied by Alfa Aesar. Zirconium chloride (ZrCl_4_), tetrafluoroterephthalic acid, 2-bromoterephthalic acid, and acetic acid were supplied by Sigma-Aldrich. 2,5-Pyridinedicarboxylic acid and 2-aminoterephthalic acid were supplied by Merck Millipore. Molecular structures and calculated p*K*_a_ values of the carboxylic linkers are shown in Figure 1.

### Synthetic Procedures

Synthetic procedures were optimized in a stepwise manner. Initially, the different Zr salts were used as the Zr source in the syntheses with the F_4_-BDC ligand at RT and at 120 °C. The salt providing the best quality of the MOF was then chosen for the synthesis with the other linkers. Finally, a study on the influence of the AcOH/H_2_O ratio on the crystallinity and surface area as a function of linker acidity was also carried out.

#### Screening of Zr Salts for the Syntheses of F_4_-UiO-66 at RT and 120 °C

Different zirconium salts (1 mmol) with 1 mmol of F_4_-BDC (238 mg) were put in an agate vessel with a 0.5 cm *ø* agate ball. The quantities were as follows: 429 mg for Zr(NO_3_)_4_·5H_2_O; 322 mg for ZrOCl_2_·8H_2_O; 233 mg for ZrCl_4_; or 303 mg for [ZrO(NO_3_)_2_·4H_2_O]. Acetic acid (AcOH, 1.0 mL, 17.5 mmol) was also added. The mixture was mechanically ground at 30 Hz for 15 min with a Fritsch planetary micro mill Pulverisette 7. The slurry was then recovered and left standing under a 25 mL beaker flipped upside down at RT for 24 h for Zr(NO_3_)_4_·5H_2_O and ZrO(NO_3_)_2_·4H_2_O. For ZrO(NO_3_)_2_·4H_2_O, the synthesis was performed at 120 °C by transferring the slurry to a 15 mL Teflon bottle for 24 h. The obtained gel was separated by centrifugation and washed three times with deionized (DI) water and once with acetone. The solid was then dried in an oven at 80 °C for 24 h. Yields: Zr(NO_3_)_4_·5H_2_O-RT = 92%; Zr(NO_3_)_4_·5H_2_O-120 = 93%; ZrOCl_2_·8H_2_O-RT = 90%; ZrCl_4_-RT = 85%; ZrO(NO_3_)_2_·4H_2_O-RT = 66%; and ZrO(NO_3_)_2_·4H_2_O-120 = 90%.

#### Syntheses of X-UiO-66 (X = NH_2_, Br, NO_2_, and Py)

For the synthesis of Br-UiO-66 and NH_2_-UiO-66, 1 mmol of Zr(NO_3_)_4_·5H_2_O (429 mg) and 1 mmol of the desired linker (181 mg for NH_2_-BDC and 245 mg for Br-BDC) were put into an agate vessel with a 0.5 cm *ø* agate ball. AcOH (0.9 mL, 15.7 mmol) and water (0.1 mL, 5.5 mmol) were added, and the mixture was mechanically ground for 15 min with a Fritsch planetary micro mill Pulverisette 7 at 50 Hz. The slurry was then transferred to a 15 mL Teflon bottle and heated at 120 °C for 24 h. The obtained gel was recovered and washed three times with DI water and once with acetone. The solid was then dried in an oven at 80 °C for 16 h. The synthesis of Br-UiO-66 was also performed by heating at 120 °C for 1 h. Yields: NH_2_-BDC-120 = 78% and Br-BDC-120 = 85%.

For the synthesis of NO_2_-UiO-66, 1 mmol of Zr(NO_3_)_4_·5H_2_O (429 mg) and 1 mmol of NO_2_-BDC (211 mg) were put into an agate vessel with a 0.5 cm *ø* agate ball; then, AcOH (1.0 mL, 17.5 mmol) was added, and the mixture was mechanically ground for 15 min with the Fritsch planetary micro mill Pulverisette 7 at 50 Hz. The slurry was then put under a beaker and left standing at RT for 24 h. The same steps described above were then carried out. Yield: 86%.

In the case of PyDC, 1 mmol of the linker (167 mg), 1 mmol of Zr(NO_3_)_4_·5H_2_O (429 mg), and 1 mL of concentrated HNO_3_ (65%, 15.57 M, 15 mmol) were added to an agate vessel with a 0.5 cm *ø* agate ball; then, the mixture was mechanically ground for 15 min with a Fritsch planetary micro mill Pulverisette 7 at 50 Hz. The resulting slurry was transferred to a 15 mL Teflon bottle and heated at 120 °C for 24 h in an oven. The same steps described above were then carried out. The same synthesis was performed by heating at 120 °C for 1 h. Yield: 88%.

#### Modulated Syntheses of X-UiO-66 (X = F_4_, NO_2_, Br, and NH_2_)

In order to investigate the modulator effect of water for the formation of clusters, various amounts of water (0–100 μL, 0–5.5 mmol) were added to an agate vessel with a 0.5 cm *ø* agate ball containing 1 mmol of Zr(NO_3_)_4_·5H_2_O (429 mg), 1 mmol of the desired linker (238 mg for F_4_-BDC, 211 mg for NO_2_-BDC, 245 mg for Br-BDC, and 181 mg for NH_2_-BDC), and AcOH (1.0–0.9 mL, 17.5–15.7 mmol) in such a way that the total volume of liquids was kept equal to 1.0 mL. The mixture was mechanically ground for 15 min with a Fritsch planetary micro mill Pulverisette 7 at 50 Hz. For the synthesis of F_4_- and NO_2_-UiO-66, the slurry was then left standing under a beaker flipped upside down at RT for 24 h, while for the synthesis of Br- and NH_2_-UiO-66, the slurry was transferred to a 15 mL Teflon bottle and heated at 120 °C for 24 h in an oven. The obtained gels were recovered and washed three times with DI water and once with acetone. The solids were then dried in an oven at 80 °C for 16 h.

#### Scaled-Up Synthesis of F_4_-UiO-66

##### Step 1

Zr(NO_3_)_4_·5H_2_O (1.96 g, 4 mmol) was ground together with 4 mmol of F_4_-BDC (0.952 g) in a mortar. AcOH (4 mL, 70 mmol) was added, and the mixture was homogenized in the mortar. The slurry was then put under a beaker and left standing at RT for 24 h. The obtained gel was separated by centrifugation and washed three times with DI water and once with acetone. The solid was then dried in an oven at 80 °C for 24 h. Yield: 1.5 g, 90%.

##### Step 2

Zr(NO_3_)_4_·5H_2_O (4.29 g, 10 mmol) was ground together with 10 mmol of F_4_-BDC (2.38 g) in a mortar. AcOH (10 mL, 175 mmol) was added, and the mixture was homogenized in the mortar. The slurry was then put under a beaker and left standing at RT for 24 h. The obtained gel was separated by centrifugation and washed three times with DI water and once with acetone. The solid was then dried in an oven at 80 °C for 24 h. Yield: 3.9 g, 93%.

### Analytical Procedures

#### Powder X-ray Diffraction

Powder X-ray diffraction (PXRD) patterns were collected with a 40 s step^–1^ counting time and with a step size of 0.016° 2θ on a PANalytical X’Pert Pro diffractometer, PW3050 goniometer, equipped with an X’Celerator detector using the Cu Kα radiation. The long fine focus ceramic tube was operated at 40 kV and 40 mA.

#### Field Emission Scanning Electron Microscopy

The morphology of the crystalline samples was investigated with a FEG LEO 1525 scanning electron microscope working with an acceleration voltage of 15 kV. Samples were preliminarily sputtered with Cr coverage to enhance the conductivity.

#### Thermogravimetric Analysis

Thermogravimetric analysis (TGA) was performed using a Netzsch STA490C thermoanalyzer under a 20 mL min^–1^ air flux with a heating rate of 10 °C min^–1^.

Nitrogen adsorption and desorption isotherms were collected using a Quantachrome Nova 2000e analyzer or a Micromeritics ASAP 2010 analyzer. Prior to the analysis, the samples were degassed overnight under dynamic vacuum at 120 °C. Brunauer–Emmett–Teller (BET) analysis and *t*-plot analysis of the adsorption data were used to calculate the specific surface area and micropore volume, respectively. Harkins and Jura equation was used as a reference for the statistical thickness calculation.

#### Nuclear Magnetic Resonance

Quantitative ^1^H and ^19^F nuclear magnetic resonance (NMR) analysis of hydrolyzed solids was performed at 25 °C on a Bruker Avance II DRX400 instrument equipped with a BBFO broadband probe. About 10–20 mg of the solid was introduced in a glass vial, which was kept in an oven at 120 °C for 2 h to remove most of the residual water from the pores. The dry solid was then weighed and treated with 1 mL of a 1 M NaOH solution in D_2_O, spiked with either 0.11 M 2-fluorobenzoic acid (for samples containing F_4_-BDC) or 0.10 M fumaric acid (for samples containing NO_2_-BDC, Br-BDC, and PyDC) as an internal standard. The mixture was briefly sonicated and left to digest overnight. The NMR tubes were then loaded with the solution, taking care to avoid transferring solid particles to the tubes. ^1^H NMR spectra were acquired by collecting four scans with an acquisition time of 5 s and *d*_1_ of 10 s. ^19^F NMR spectra were acquired by collecting four scans with an acquisition time of 3 s and *d*_1_ of 5 s.

## Results and Discussion

We started our investigation by screening different commercial Zr^IV^ precursors in combination with F_4_-BDC, which was demonstrated by Huang *et al.*(31) to be very prone to rapidly form a UiO-66 phase when milled with preformed hexanuclear Zr^IV^ clusters. The syntheses were carried out by ball milling equimolar amounts of the Zr^IV^ precursor and F_4_-BDC (1 mmol each) in a planetary ball mill with a 0.5 cm *ø* agate ball in the presence of 1.0 mL of AcOH for 15 min. The resulting slurry was then transferred to a closed container and incubated at RT for 24 h.

**Figure 2:**
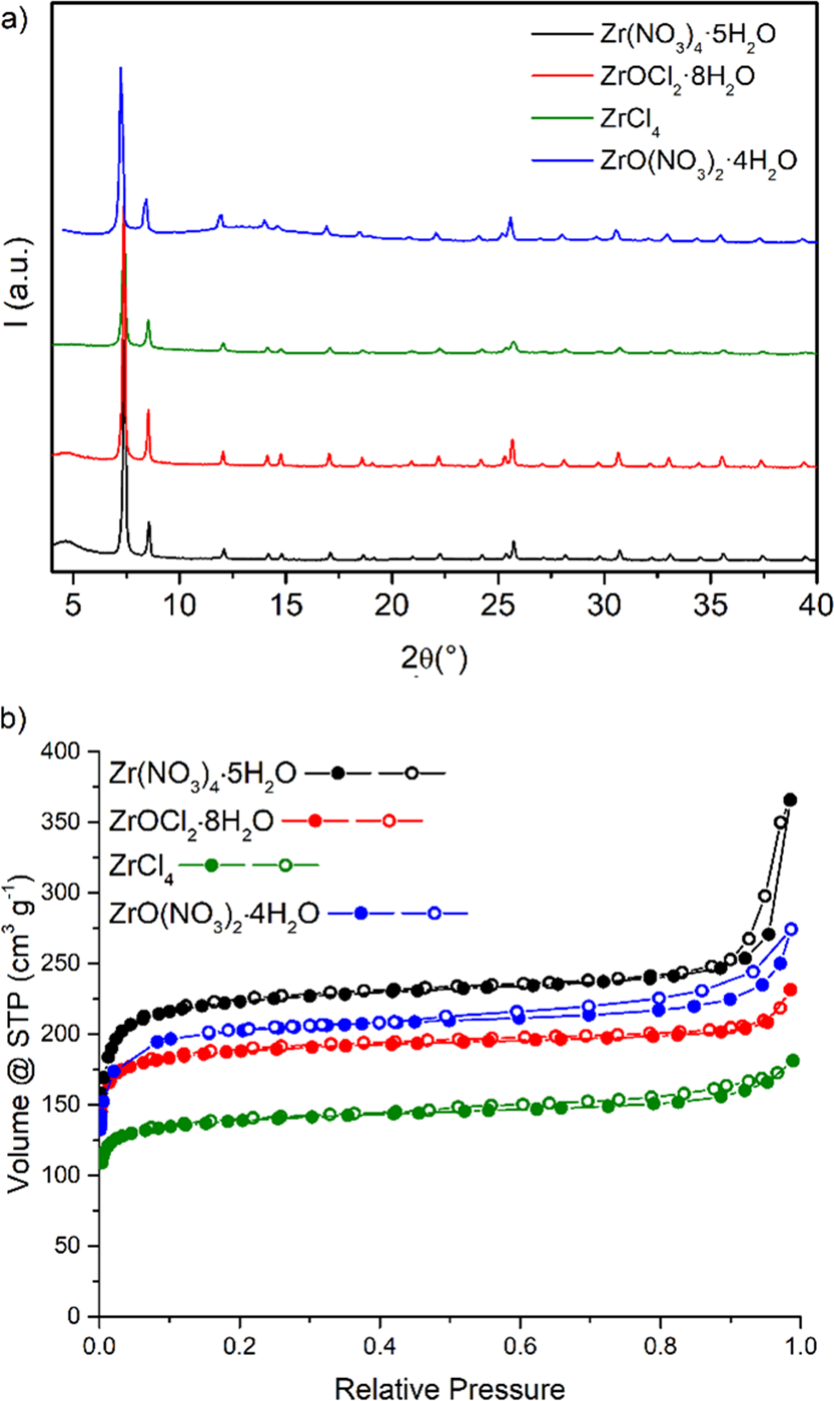
PXRD patterns (a) and N_2_ adsorption isotherms at 77 K (b) of the products obtained from the reaction of F_4_-BDC with Zr(NO_3_)_4_·5H_2_O (black), ZrOCl_2_·8H_2_O (red), and ZrCl_4_ (olive) at RT and with ZrO(NO_3_)_2_·4H_2_O at 120 °C (blue).

PXRD patterns were collected *ex situ* just after the milling process and after 3, 5, and 24 h in order to evidence the efficacy of the aging process (Figure S1). The fresh mixture already presented two reflections similar to the 111 and 200 of the UiO-66 **fcu** phase, although the position was slightly displaced. Peaks belonging to the linker are also present, whereas those relative to the UiO phase slightly change in their position. After 24 h, the PXRD pattern shows the formation of a well-crystallized UiO-66-like phase. The mixture after 24 h was washed with water and acetone to remove every possible unreacted reagent and centrifuged to recover the solid. Using these amounts of reagents and AcOH, a concentration of 1 M of both the salt and linker was obtained, which is 10 to 40 times higher than that normally used for DMF- or water-based syntheses of UiO-66-type MOFs.^32,37,41−43^ We obtained phase-pure and crystalline UiO-66 from Zr(NO_3_)_4_·5H_2_O, ZrOCl_2_·8H_2_O, and ZrCl_4_ at RT (Figure 2a). In the case of ZrO(NO_3_)_2_·4H_2_O, the mixture had to be heated to 120 °C in order to obtain a crystalline product (Figure S2). The products obtained from Zr(NO_3_)_4_·5H_2_O and ZrOCl_2_·8H_2_O displayed broad reflections around 4–5° 2θ, which could be associated with either the presence of regions of missing-cluster defects with the **reo** topology^44,45^ or the presence of a phase displaying **hcp** topology.^46^ No residual reflections of the linker were present in the PXRD patterns of the products, proving the efficacy of the workup procedure (Figure S3). Scanning electron microscopy (SEM) micrographs show that MOF crystallites with ill-defined morphology and size in the nanometric range (below 100 nm) were formed (Figure S4). The use of ZrCl_4_ afforded MOF with slightly lower yield and a lower degree of crystallinity and surface area with respect to the other precursors, which could be due to the lack of crystallization water in this salt. Given that 1.33 equiv of water is needed to provide the oxide and hydroxide ions constituting the metal clusters, water is essential for the successful formation of the MOF. The fact that we still obtained the MOF using ZrCl_4_ suggests that the precursor did contain some water, due to its strong tendency to absorb humidity from the atmosphere, but the amount was not stoichiometric. The N_2_ adsorption–desorption isotherms at 77 K obtained with these samples are reported in Figure 2b, and they can be classified as type I isotherms, which are typical of microporous materials. The specific surface area and micropore volumes were calculated from the BET and *t*-plot analyses of the adsorption data, respectively, and are reported in Table 1. The highest BET surface area of 802 m^2^ g^–1^ was recorded for the sample obtained from Zr(NO_3_)_4_·5H_2_O. This value is higher than that previously reported for perfluorinated UiO-66 synthesized in water.^32,36^

**Table 1:** BET Surface Area and Micropore Volume Values for Perfluorinated UiO-66 Samples Synthesized Starting from Different Zr Precursors

precursor	incubation temperature	BET surface area (m^2^ g^–1^)	micropore volume (cm^3^ g^–1^)
Zr(NO_3_)_4_·5H_2_O	RT	802	0.30
ZrOCl_2_·8H_2_O	RT	750	0.27
ZrCl_4_	RT	540	0.19
ZrO(NO_3_)_2_·4H_2_O	120 °C	783	0.30

TGA showed that all products start decomposing at a similar temperature of 300 °C, consistent with what is observed in previous literature reports (Figure S5).^1,36^ These results suggest that Zr(NO_3_)_4_·5H_2_O is the most suitable precursor to obtain F_4_-UiO-66 with high crystallinity and porosity under mild conditions.

**Figure 3:**
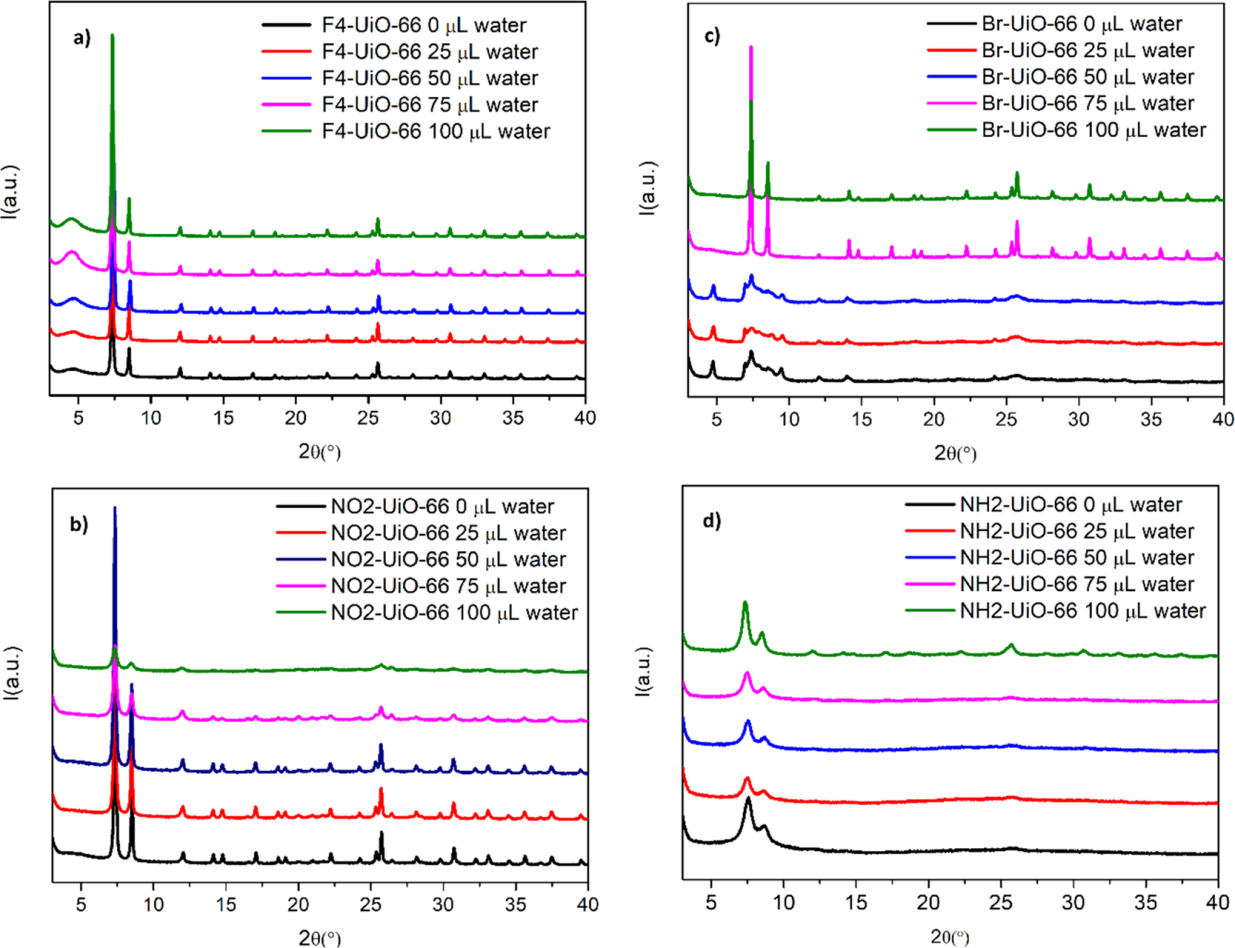
PXRD patterns of (a) F_4_-UiO-66, (b) NO_2_-UiO-66, (c) Br-UiO-66, and (d) NH_2_-UiO-66 synthesized by changing the amount of water: 0 μL (black lines), 25 μL (red lines), 50 μL (blue lines), 75 μL (purple lines), and 100 μL (green lines).

We then moved on to screen linkers bearing different functional groups. The same synthetic procedure described above was employed, using Zr(NO_3_)_4_·5H_2_O as the metal precursor and replacing F_4_-BDC with either NO_2_-BDC, Br-BDC, NH_2_-BDC, BDC, or PyDC. However, a different behavior, apparently depending on the acidity, was observed for these linkers. Highly acidic linkers containing electron-withdrawing groups (such as F_4_-BDC and NO_2_-BDC) afforded well-crystallized MOFs in pure AcOH, whereas less acidic linkers containing electron-donating groups (Br-BDC and NH_2_-BDC) were not able to successfully react in pure AcOH and needed a small amount of water in the synthesis to yield highly crystalline products. In order to gather additional insights into this aspect, we performed a systematic screening of the influence of the AcOH/H_2_O ratio in the crystallinity and porosity of the obtained products. The syntheses were then performed by changing the amount of water from 0 μL (1 mL of pure AcOH) to 100 μL (900 μL of AcOH/100 μL of H_2_O) with intermediate values (25, 50, and 75 μL). PXRD patterns, TGA analysis, and N_2_ adsorption isotherms of the samples obtained under different synthetic conditions are shown in Figures S5 and S6. Figure 3 shows the PXRD patterns of F_4_- (a), NO_2_- (b), Br- (c), and NH_2_- (d) UiO-66 MOFs obtained at different water/AcOH ratios, whereas the BET surface area and micropore volumes are reported in Table 2.

**Table 2:** BET Surface Area and Micropore for UiO-66 Samples Synthesized with Different Amounts of Water, in Addition to AcOH

sample	amount of water (μL)	BET surface area (m^2^ g^–1^)	BET surface area from the literature (solvothermal syntheses) (m^2^ g^–1^)	micropore volume (cm^3^ g^–1^)
F_4_-UiO-66	0	802	690^50^	0.305
	50	719		0.283
	100	713		0.270
NO_2_-UiO-66	0	793	756^48^	0.303
	25	758		0.292
	50	630		0.242
Br-UiO-66	100	724	851^48^	0.282
NH_2_-UiO-66	100	519	1112^48^	0.201

sample	solvent	BET surface area (m^2^ g^–1^)	BET surface area from the literature (solvothermal syntheses) (m^2^ g^–1^)	micropore volume (cm^3^ g^–1^)
Py-UiO-66	HNO_3_ 65%, 1 mL	979	1380^51^	0.361

F_4_-UiO-66 crystallization seems to be little affected by the water/AcOH ratio, as all the PXRD patterns are indicative of highly crystalline compounds. An increase in the intensity of the broad reflection at a low 2θ angle is observed at higher water amounts, which could indicate the presence of some missing-cluster defects. However, BET surface areas and micropore values appear to slightly decrease from 802 to 713 m^2^ g^–1^ as the water amount increases, which is not the expected effect for defective UiO-66-type MOFs.^45^ It was recently demonstrated that F_4_-UiO-66 can also crystallize in a hexagonal form with **hcp** topology, which also features a reflection in the region around 5° 2θ and that the amount of this defective phase can be modulated using water in the solvothermal synthesis.^46^ While missing-cluster defects are expected to increase the porosity of the framework, the **hcp** phase is less porous; therefore, our results—showing a decrease in porosity associated with the increase in the amount of water used in the synthesis—seem to suggest that we are likely forming a small amount of the **hcp** phase as an impurity. Quantitative ^1^H and ^19^F NMR analysis of the digested MOFs (Figures S7–S9) leads to determine the same chemical formula for samples prepared with 0, 50, and 100 μL of water, that is, Zr_6_O_4_(OH)_4_(F_4_-BDC)_5.73_(AcOH)_0.54_. The very low amount of AcOH retained in the framework suggests that a very small amount of defects is present in fact, further supporting the hypothesis that the broad reflection at a low angle in the PXRD pattern can be assigned to the **hcp** phase.

In the case of NO_2_-UiO-66, the trend is similar to F_4_-UiO-66, although the addition of 75 to 100 μL of water leads to a more evident loss of crystallinity and porosity with respect to the samples obtained either in pure AcOH or with a lower amount of water (25–50 μL). The BET surface area of the sample obtained in pure AcOH is 793 m^2^ g^–1^, in line with values found in the literature. The addition of 25 μL of water leads to a small decrease to 758 m^2^ g^–1^, whereas upon the addition of 50 μL of water, the value drops to 630 m^2^ g^–1^. Quantitative ^1^H NMR analysis (Figures S10–S12) suggests that samples prepared with 0, 25, and 50 μL of water contain small amounts of defects, with the representative formula Zr_6_O_4_(OH)_4_(NO_2_-BDC)_5.75_(AcOH)_0.50_. However, the discrepancy between the calculated mass composition based on the abovementioned formula and the experimental one derived from the absolute amounts through the use of an internal standard suggests that the products contain some impurity, possibly of inorganic nature, and that the amount of such impurity increases with increasing amount of water used in the synthesis, thus accounting for the observed drop in the BET surface area.

Concerning Br- and NH_2_-UiO-66, the trend observed so far is reversed: the products with higher crystallinity are those resulting from syntheses in the presence of a higher water quantity. This effect is more pronounced for Br-UiO-66, where the samples obtained with less than 75 μL of water display a different PXRD pattern from that of UiO-66, whereas samples obtained with 75 and 100 μL of water are highly crystalline, and the BET surface area (724 m^2^ g^–1^) is consistent with that reported for conventional syntheses in DMF.^41^ In this case, quantitative ^1^H NMR analysis (Figure S13) reveals the presence of a significant amount of AcOH and the proposed formula is Zr_6_O_4_(OH)_4_(Br-BDC)_5.06_(AcOH)_1.88_. Finally, NH_2_-UiO-66 was obtained in low crystallinity in all the syntheses, with a slight improvement for the sample made with 100 μL of water. Furthermore, the porosity of this product was much lower than that of the MOF obtained from a conventional synthesis in DMF.^41,43,47,48^ Compared to the other linkers, NH_2_-BDC is by far the one less able to afford MOF of high quality. We also attempted syntheses employing bare BDC as the linker, consistently obtaining amorphous products, regardless of the amount of water used.

We speculate that the different behavior displayed by different linkers is probably attributed to their acidity. As shown in Figure 1, p*K*_a_1__ values of BDC and NH_2_-BDC are 3.32 and 4.03, respectively, about 1 order of magnitude lower than that of Br-BDC (2.72) and more than 2 orders of magnitude lower than those of F_4_-BDC (1.18) and NO_2_-BDC (0.72). The effect can be twofold: (1) BDC and NH_2_-BDC are not able to effectively deprotonate under reaction conditions, that is, in the presence of an excess of a mild acid such as AcOH (p*K*_a_ = 4.64) and (2) since solubility in water is highly dependent on the acidity of the carboxylic linker, which is in turn related to the presence of electron-withdrawing substituents on the aromatic ring, we speculate that less acidic linkers, such as BDC and NH_2_-BDC, fail to dissolve in the small amount of water contained in the Zr precursor, thus preventing crystallization of the MOF from occurring. On the other hand, the higher water solubility of F_4_-BDC, NO_2_-BDC, and Br-BDC allows the rapid reaction with hydrated Zr salts upon grinding and successive incubation at RT or 120 °C.

A different situation was encountered when the synthesis of Py-UiO-66 was attempted, which afforded nearly amorphous products using AcOH at both RT and 120 °C. We note that this linker features a highly acidic carboxylate (p*K*_a_1__ = 0.80) and a pyridinic N atom. As a consequence, PyDC is the only linker, among those tested here, able to form a zwitterion through proton transfer from the carboxylate to the pyridinic N atom. Thus, the presence of a deprotonated carboxylate makes it very prone to coordinate to the metal, with very fast crystallization kinetics that lead to a poorly crystalline product. Therefore, according to a previous study where a large excess of concentrated HCl was employed to induce crystallization of Py-UiO-66,^49^ we decided to use concentrated HNO_3_ (65 wt %) in place of AcOH. We chose to use HNO_3_ instead of HCl to avoid the introduction of additional species, that is, chloride, in the reaction mixture. This strategy afforded a product with improved quality, but still not completely satisfactory (Figure S13). Given the harsh conditions in which this synthesis is performed, we speculated that prolonged heating could harm the product; therefore, we attempted to reduce the reaction time down to 1 or 2 h. In both cases, highly crystalline products were recovered (Figure S13). The ^1^H NMR spectra of the digested sample confirmed the purity of the compound and the possible presence of nitrates into the structure (Figure S14). Since the HNO_3_ solution already contains a high amount of water, water screening was not carried out in this case.

The positive results obtained upon reduction of the reaction time with Py-UiO-66 prompted us to investigate if also other compounds could be obtained in a shorter time than the initially employed 24 h. We chose the most crystalline sample of every set of compounds (F_4_-UiO-66, 0 μL, NO_2_-UiO-66, 25 μL, and Br-UiO-66, 100 μL) and reduced the reaction time down to 1 and 2 h. The PXRD patterns of Figure S15 demonstrate the formation of highly crystalline Br-UiO-66 also after 1 or 2 h, while F_4_-UiO-66 and NO_2_-UiO-66 can be achieved but display low crystallinity. This is likely due to the slower crystallization kinetics for the syntheses carried out at RT, which require a longer time to reach completion.

To check the possibility of obtaining good quality compounds on a larger scale, the synthesis of F_4_-UiO-66 was scaled-up in two steps. The synthesis was initially attempted using 4 mmol each of F_4_-BDC and Zr(NO_3_)_4_·5H_2_O and 4 mL of AcOH by milling for 1 h at 30 Hz, observing the formation of a UiO-66 phase with low crystallinity and porosity (Figures S16a and S17a). In a successive attempt, where the vessel was kept sealed for 24 h after initial milling, 1.5 g of a well-crystallized compound was obtained, whose crystallinity is comparable to that obtained using 1 mmol of reagents (Figure S15b). This product displays a BET s.a. of 888 m^2^ g^–1^ and micropore volume of 0.33 cm^3^ g^–1^, about the same as that measured for the MOF obtained with the small-scale synthesis (Figure S16b). Finally, a last synthesis employing 10 mmol of reagents [F_4_-BDC and Zr(NO_3_)_4_·5H_2_O] was carried out by just mixing them in a mortar and bypassing the milling step (see Experimental Section). Almost 4 g of good quality MOF (Figure S18) was obtained, demonstrating the easy scalability of the procedure up to a 10-fold scale.

## Conclusions

We have reported a simple “shake ‘n bake” procedure for the synthesis of a range of functionalized Zr-based UiO-66 MOFs starting from commercial Zr^IV^ precursors. The method involves mixing of the metal salt, linker, and a liquid reagent in a ball mill, followed by incubation of the mixture at either RT or 120 °C. We demonstrated the efficacy of the procedure for the synthesis when using functionalized carboxylic ligands with higher acidity and water solubility than simple BDC. The syntheses are quick and afford pure compounds with comparable crystallinity and porosity as those obtained by conventional solvothermal synthesis. Scalability was also proven to be effective up to 10-fold. This method allows us to avoid toxic solvents and adds up to the range of the existing approaches for the sustainable synthesis of MOFs for industrial applications.

## Supporting Information Available

PXRD patterns of MOFs and linkers, TGA curves, SEM images, N_2_ adsorption and desorption isotherms, ^1^H NMR spectra, PXRD patterns, and BET curves for scale-up syntheses (PDF)
