# Supplementary material for: “Shake ‘n Bake” Route to Functionalized Zr-UiO-66 Metal–Organic Frameworks
Source: Inorg Chem. 2021 Sep 2;60(18):14294–301. doi: 10.1021/acs.inorgchem.1c01839 (PMC8456408; doi:10.1021/acs.inorgchem.1c01839)
Supplement: Supplementary file 1 — ic1c01839_si_001.pdf [file ic1c01839_si_001.pdf]

A "Shake 'n Bake" Route to Functionalized Zr-UiO-66 Metal-Organic Frameworks

Roberto D'Amato,[1,2] Roberto Bondi,[1] Intissar Moghdad,[3] Fabio Marmottini,[1] Matthew J. McPherson,[4] Houcine Naïli,[5] Marco Taddei[4,6,*] and Ferdinando Costantino[1,*]

[1]Dipartimento di Chimica Biologia e Biotecnologia, University of Perugia, Via Elce di Sotto 8, 06123 Perugia, Italy. Email: ferdinando.costantino@unipg.it

[2]International Iberian Nanotechnology Laboratory, Avenida Mestre José Veiga s/n, 4715-330 Braga, Portugal.

[3]Laboratory of Advanced Materials, National Engineering School, Sfax University, P.B. 1173, 3038 Sfax, Tunisia

[4]Energy Safety Research Institute, Swansea University, Fabian Way, Swansea SA1 8EN, United Kingdom.

[5]Laboratory Physico Chemistry of the Solid State, Department of Chemistry, Faculty of Sciences of Sfax, P.B. 1171, 3000 Sfax, Sfax University, Tunisia.

[6]Dipartimento di Chimica e Chimica Industriale, Università di Pisa, Via Giuseppe Moruzzi, 13, 56124 Pisa, Italy. Email: marco.taddei@unipi.it

Supporting Information

1-PXRD PATTERNS

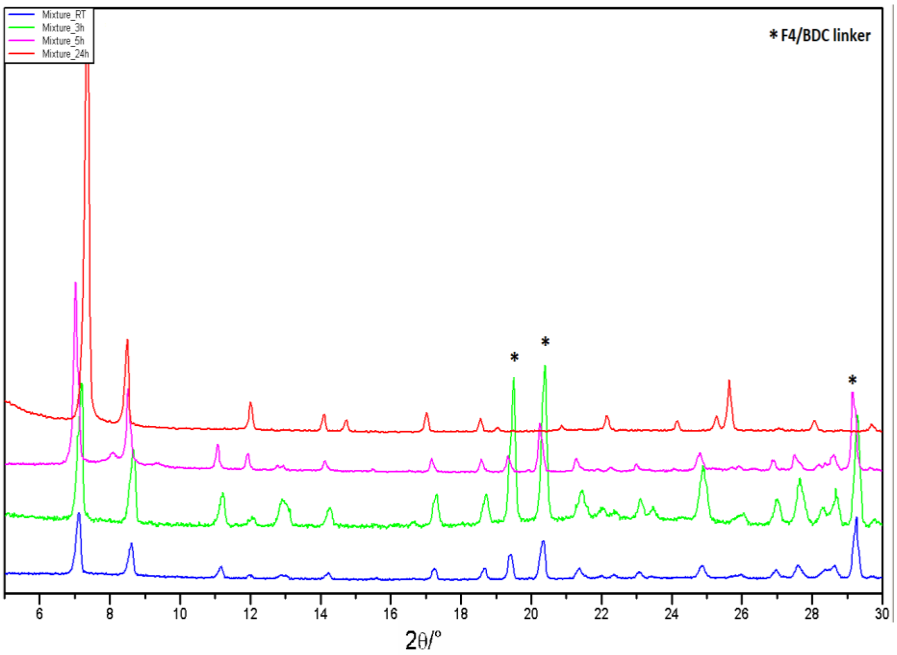

Figure S1. XRPD patterns of the mixture after ball milling recorded immediately (blue line) and after 3, 5 and 24h (green, violet and red lines) respectively

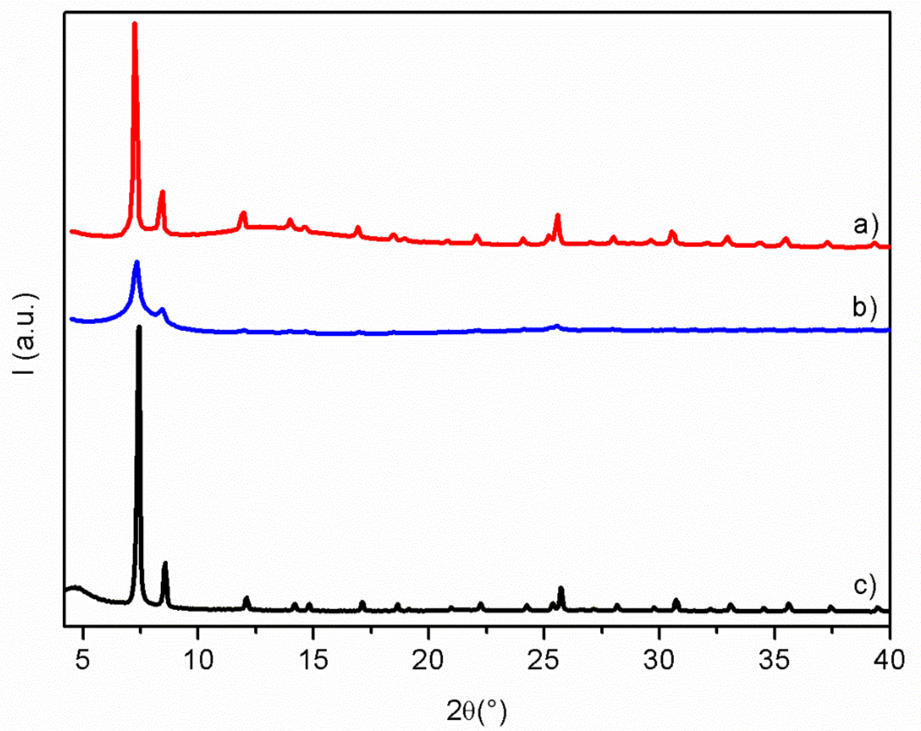

Figure S2. PXRD pattern of F_4-UiO-66 from $ZrO(NO_3)_2{\cdot}4H_2O$ at 120 °C (a) and PXRD pattern of F_4-UiO-66 from $ZrO(NO_3)_2{\cdot}4H_2O$ at RT (b) compared with the pattern of F4-UiO-66 obtained from $Zr(NO_3)_4{\cdot}5H_2O$ at RT (c).

2- PXRD PATTERNS RESIDUAL LINKERS

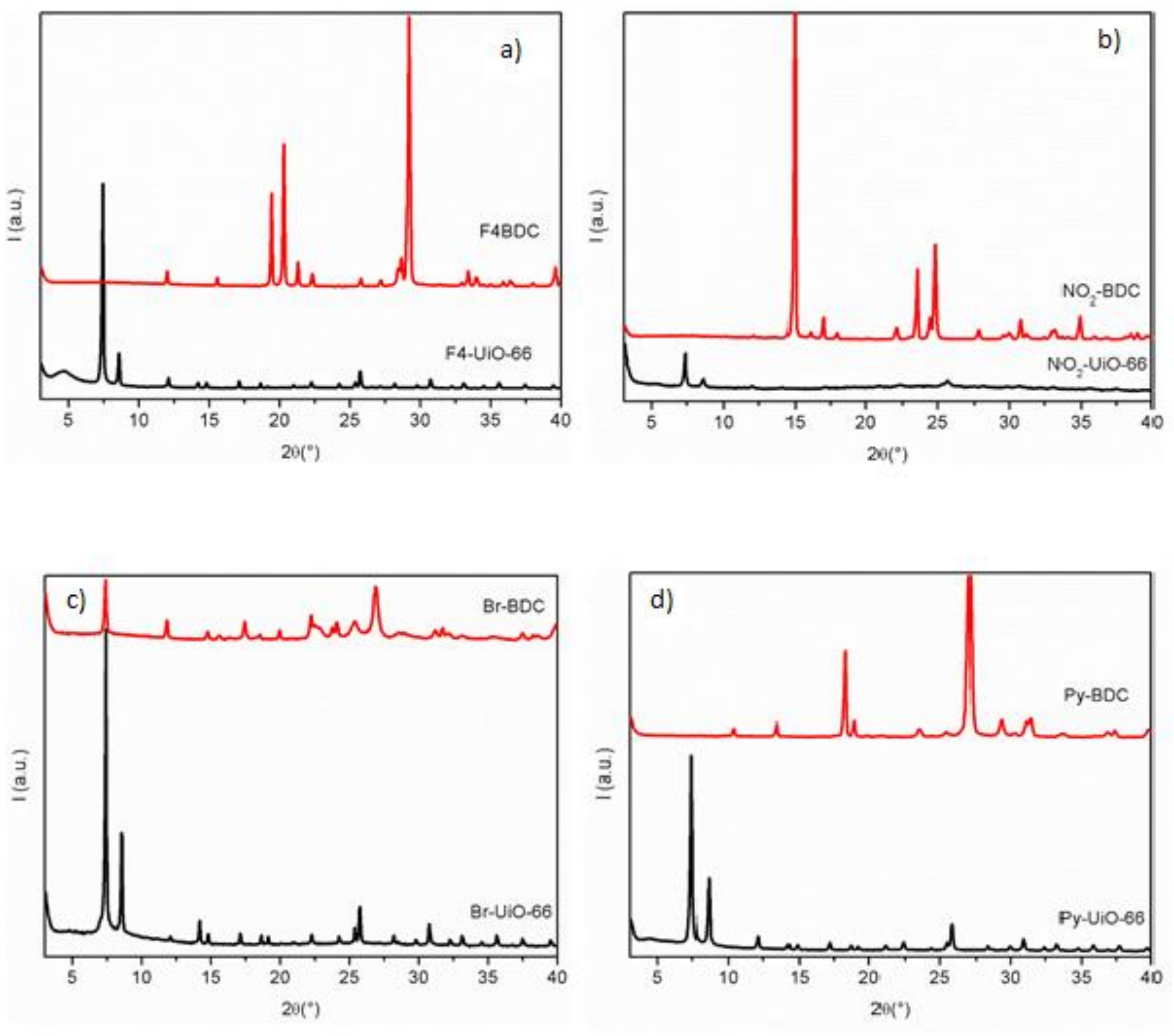

Figure S3. Comparison of PXRD patterns of the MOFs with relative linkers. a) F_4-UiO-66 and F_4-BDC; b) NO_2-UiO-66 and NO_2-BDC; c) Br-UiO-66 and Br-BDC; d) Py-UiO-66 and PyDC. No peaks of residual linkers can be detected after washing.

3-SEM IMAGES

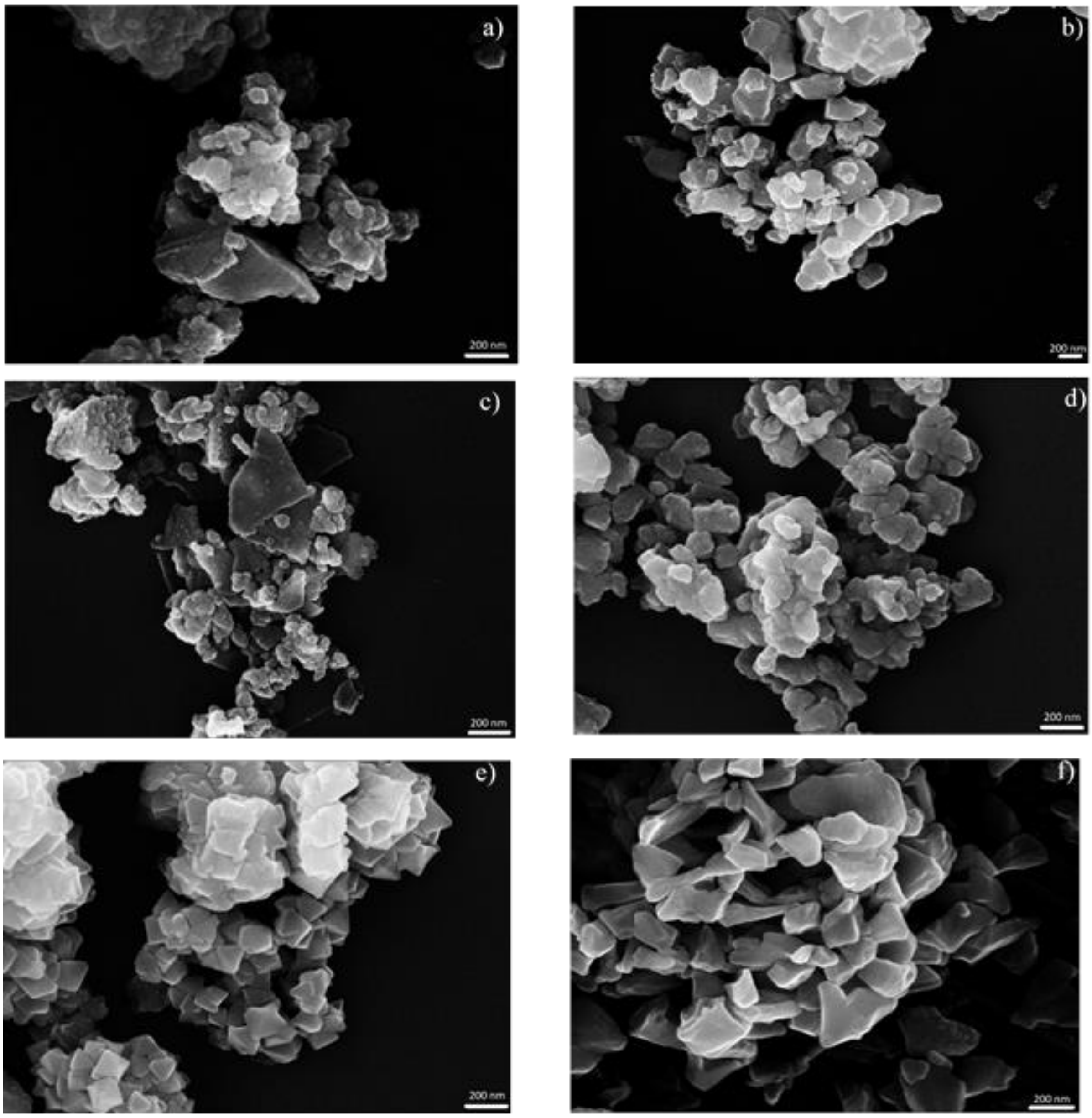

Figure S4. SEM images of: a) F_4-UiO-66-$Zr(NO_3)_4$, b) F_4-UiO-66-$ZrOCl_2$, c) F_4-UiO-66-$ZrCl_4$, d) NO_2-UiO-66, e) Py-UiO-66, f) Br-UiO-66.

4-TGA CURVES

Figure S5. TGA curves for a) F4-UiO-66 0, 50, 100 µL water; b) NO_2-UiO-66 0, 50, 100 µL water; c) Br-UiO-66 100 µL water; d) Py-UiO-66 and e) NH_2-UiO-66 100 µL water

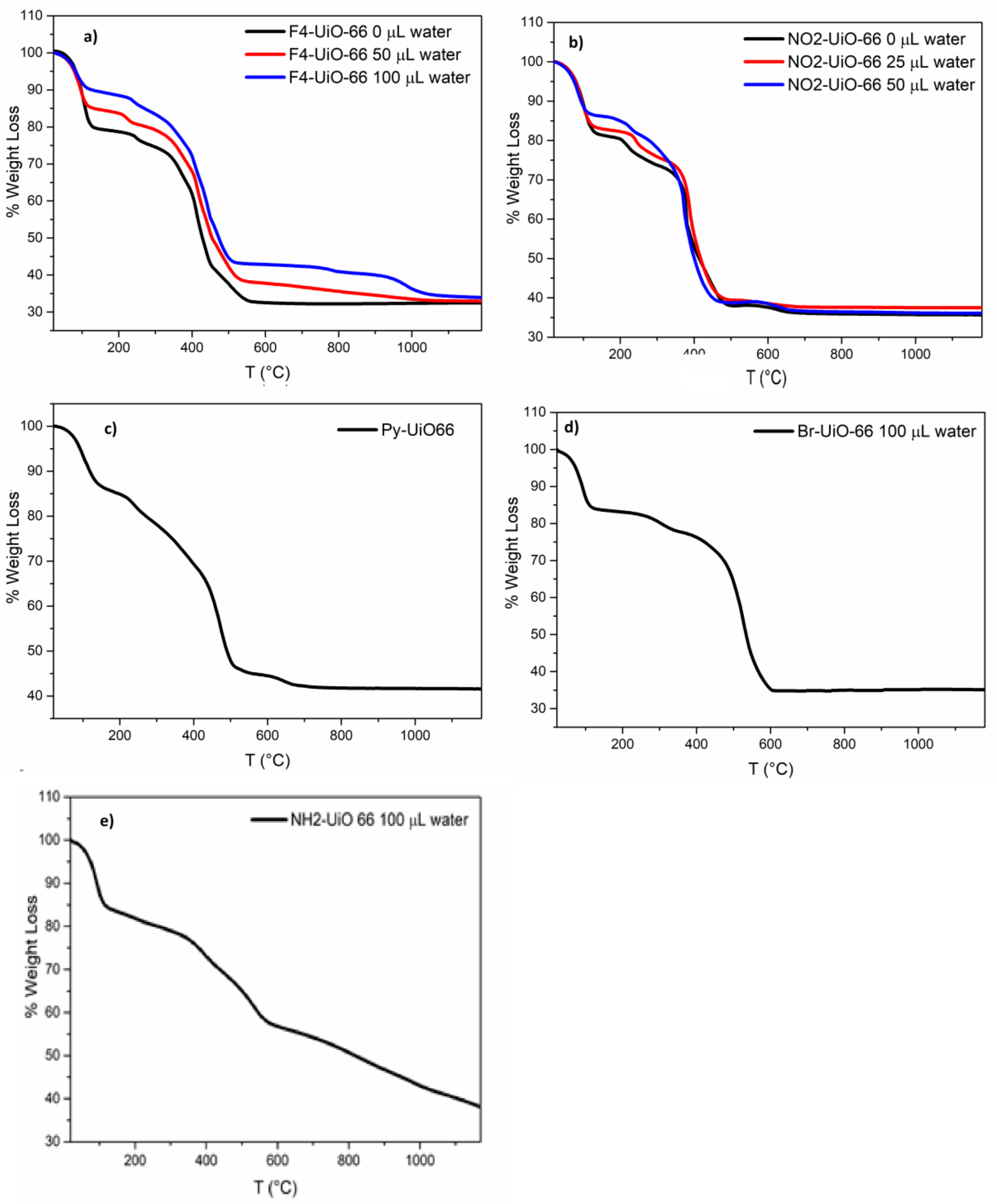
a)
F4-UiO-66 0 μL water
F4-UiO-66 50 μL water
F4-UiO-66 100 μL water
% Weight Loss
T (°C)
b)
NO2-UiO-66 0 μL water
NO2-UiO-66 25 μL water
NO2-UiO-66 50 μL water
% Weight Loss
T (°C)
c)
Py-UiO66
% Weight Loss
T (°C)
d)
Br-UiO-66 100 μL water
% Weight Loss
T (°C)
e)
NH2-UiO 66 100 μL water
% Weight Loss
T (°C)

5-N_2 adsorption/desorption isotherms

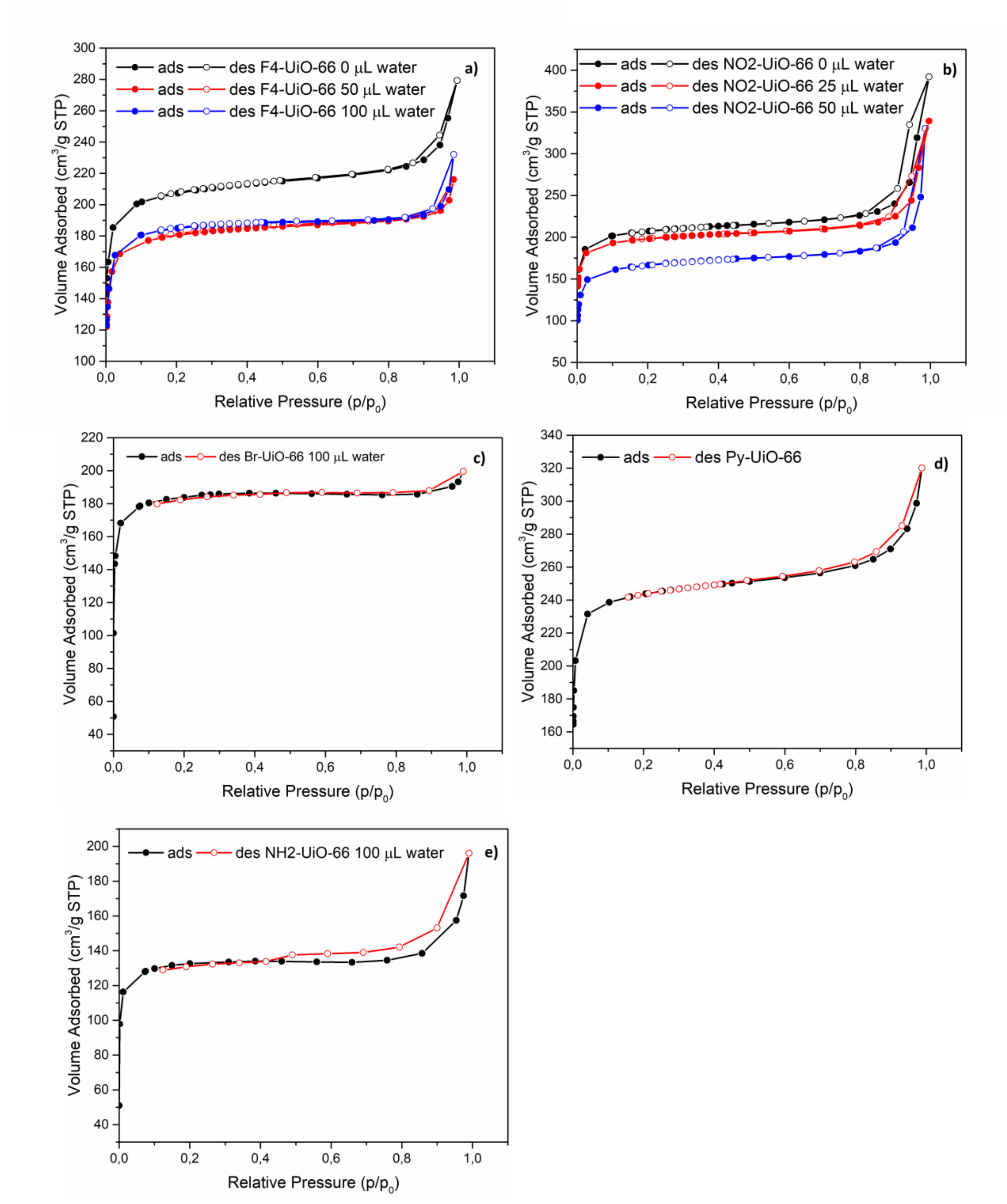

Figure S6. N_2 adsorption/desorption isotherms for a) F4-UiO-66 0, 50, 100 μL water; b) NO_2-UiO-66 0, 50, 100 μL water; c) Br-UiO-66 100 μL water; d) Py-UiO-66 and e) NH_2-UiO-66 100 μL water.

6-NMR SPECTRA

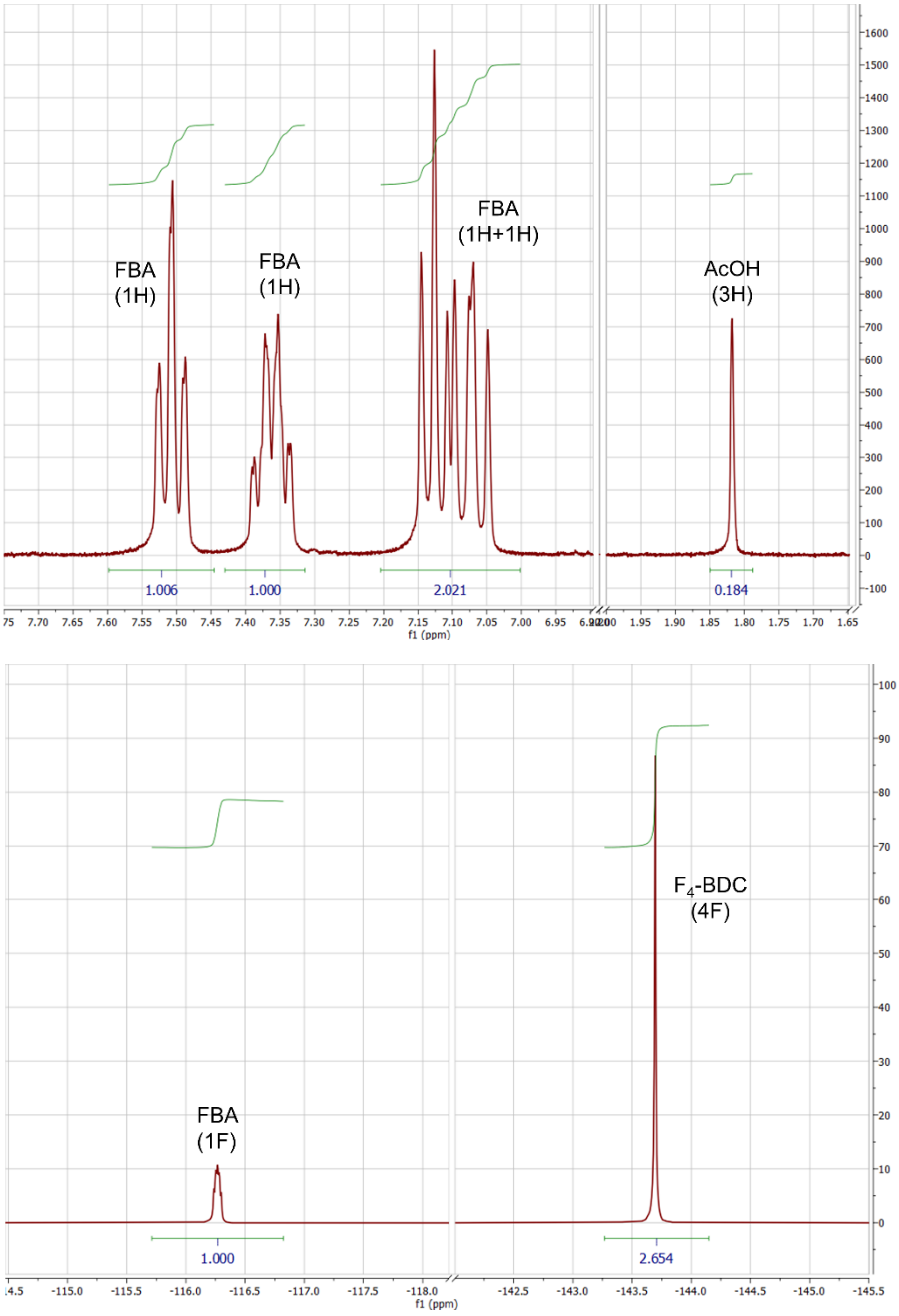

Figure S7. ^{1}H (top) and ^{19}F (bottom) NMR spectra of F_4-UiO-66 0 μL water.

25.9 mg of desolvated MOF was digested. In the ^{1}H NMR spectrum, the four signals of internal standard 2-fluorobenzoic acid (FBA) fall between 7.00 and 7.60 ppm and account for one proton each. The signal of AcOH falls at 1.82 ppm and accounts for three protons. Thus, in order to have a quantitative comparison of the two species, the integral of AcOH (0.184) must be divided by three, giving 0.061. In the ^{19}F NMR spectrum, the signal of

internal standard FBA falls at -116.2 ppm and accounts for one proton. The signal of F_4-BDC falls at -143.7 ppm and accounts for four protons. Thus, in order to have a quantitative comparison of the two species, the integral of F_4-BDC (2.654) must be divided by four, giving 0.664. This leads to calculate a F_4-BDC/AcOH ratio of 10.9. Assuming the general formula $Zr_6O_4(OH)_4(F_4\text{-BDC})_{6-x}(AcOH)_{2x}$, the following can be written:

$$\frac{6-x}{2x} = 10.9 \qquad \text{(equation } \mathbf{S1}\text{)}$$

Solving equation **S1**, a value of 0.26 for *x* is determined, leading to the following proposed formula: $Zr_6O_4(OH)_4(F_4\text{-BDC})_{5.74}(AcOH)_{0.52}$, having formula weight (FW) of 2063 g mol^{-1}.

The absolute concentration of AcOH in solution is obtained by multiplying its normalised integral (0.061) times the concentration of FBA (0.11 M), obtaining 0.0067 M. The absolute amount (in mg) of AcOH in the MOF can be derived by multiplying the concentration times volume of the solution (1.0 mL) and the molecular weight of the acetate anion (59 g mol^{-1}), obtaining 0.40 mg. This leads to derive an experimental wt% of 1.54% for AcOH in the desolvated MOF. The absolute concentration of F_4-BDC in solution is obtained by multiplying its normalised integral (0.664) times the concentration of FBA (0.11 M), obtaining 0.0730 M. The absolute amount (in mg) of F_4-BDC in the MOF can be derived by multiplying the concentration times volume of the solution (1.0 mL) and the molecular weight of the F_4-BDC dianion (236 g mol^{-1}), obtaining 17.22 mg. This leads to derive an experimental wt% of 66.50% for F_4-BDC in the desolvated MOF. According to the proposed formula $Zr_6O_4(OH)_4(F_4\text{-BDC})_{5.74}(AcOH)_{0.52}$, the calculated wt% of AcOH is 1.49%, while that of F_4-BDC is 65.65%. The good agreement between experimental and calculated wt% suggests that the proposed formula is correct and that the analysed MOF does not contain impurities.

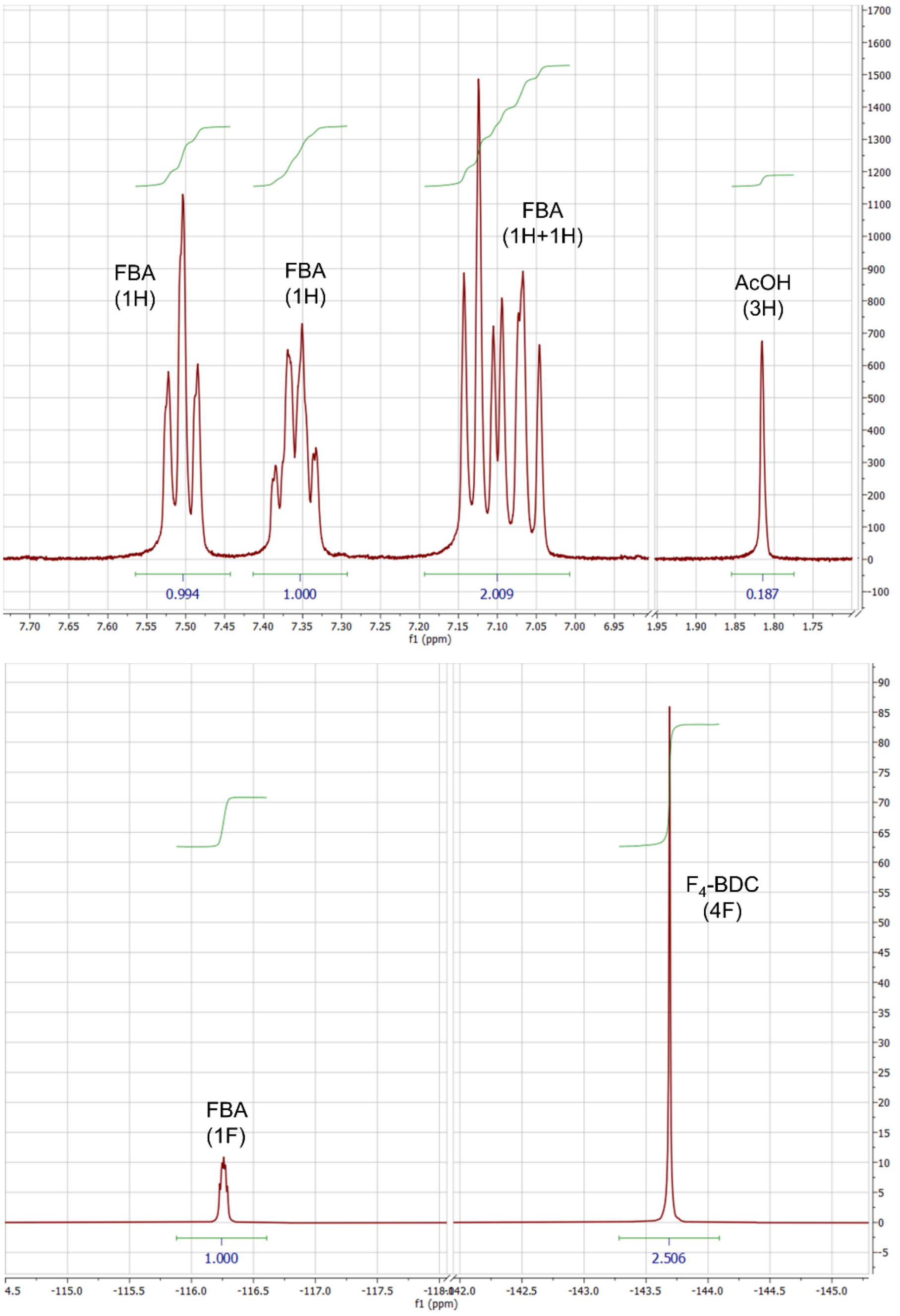

Figure S8. ^{1}H (top) and ^{19}F (bottom) NMR spectra of F_4-UiO-66 50 μL water.

24.8 mg of desolvated MOF was digested. In the ^{1}H NMR spectrum, the four signals of internal standard 2-fluorobenzoic acid (FBA) fall between 7.00 and 7.60 ppm and account for one proton each. The signal of AcOH falls at 1.82 ppm and accounts for three protons. Thus, in order to have a quantitative comparison of the two species, the integral of AcOH (0.187) must be divided by three, giving 0.062. In the ^{19}F NMR spectrum, the signal of internal standard FBA falls at -116.2 ppm and accounts for one proton. The signal of F_4-BDC falls at -143.7 ppm and accounts for four protons. Thus, in order to have a quantitative

comparison of the two species, the integral of F_4-BDC (2.506) must be divided by four, giving 0.627. This leads to calculate a F_4-BDC/AcOH ratio of 10.1. Assuming the general formula $Zr_6O_4(OH)_4(F_4\text{-BDC})_{6-x}(AcOH)_{2x}$, the following can be written as the same way of eq. **S1**:

$$\frac{6-x}{2x} = 10.1 \qquad \text{(equation } \mathbf{S2}\text{)}$$

Solving this equation, a value of 0.28 for *x* is determined, leading to the following proposed formula: $Zr_6O_4(OH)_4(F_4\text{-BDC})_{5.72}(AcOH)_{0.56}$, having FW of 2061 g mol^{-1}.

The absolute concentration of AcOH in solution is obtained by multiplying its normalised integral (0.062) times the concentration of FBA (0.11 M), obtaining 0.0069 M. The absolute amount (in mg) of AcOH in the MOF can be derived by multiplying the concentration times volume of the solution (1.0 mL) and the molecular weight of the acetate anion (59 g mol^{-1}), obtaining 0.40 mg. This leads to derive an experimental wt% of 1.63% for AcOH in the desolvated MOF. The absolute concentration of F_4-BDC in solution is obtained by multiplying its normalised integral (0.627) times the concentration of FBA (0.11 M), obtaining 0.0689 M. The absolute amount (in mg) of F_4-BDC in the MOF can be derived by multiplying the concentration times volume of the solution (1.0 mL) and the molecular weight of the F_4-BDC dianion (236 g mol^{-1}), obtaining 16.26 mg. This leads to derive an experimental wt% of 65.58% for F_4-BDC in the desolvated MOF. According to the proposed formula $Zr_6O_4(OH)_4(F_4\text{-BDC})_{5.72}(AcOH)_{0.56}$, the calculated wt% of AcOH is 1.60%, while that of F_4-BDC is 65.50%. The good agreement between experimental and calculated wt% suggests that the proposed formula is correct and that the analysed MOF does not contain impurities.

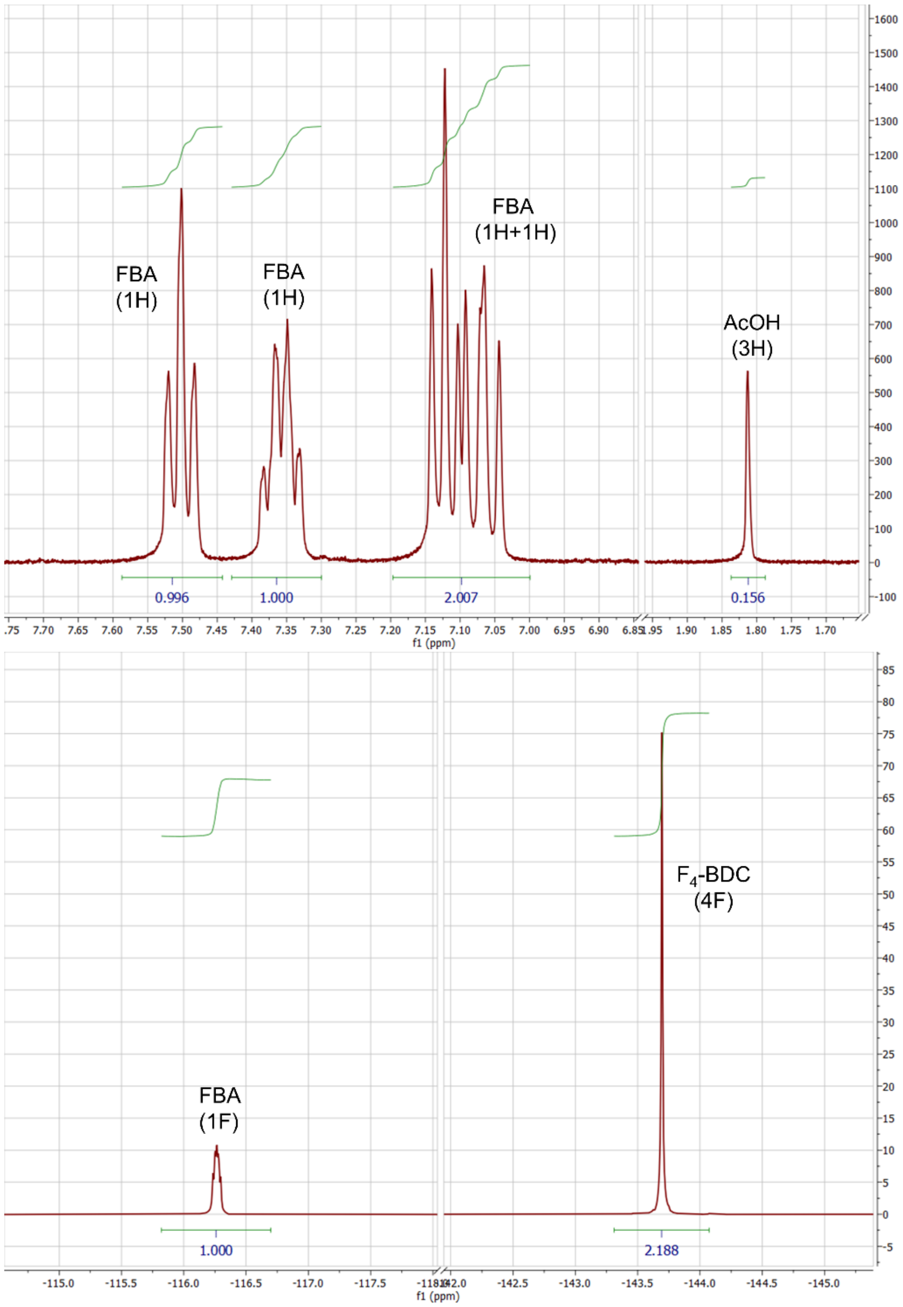

Figure S9. ^{1}H (top) and ^{19}F (bottom) NMR spectra of F_4-UiO-66 100 μL water.

21.2 mg of desolvated MOF was digested. In the ^{1}H NMR spectrum, the four signals of internal standard 2-fluorobenzoic acid (FBA) fall between 7.00 and 7.60 ppm and account for one proton each. The signal of AcOH falls at 1.82 ppm and accounts for three protons. Thus, in order to have a quantitative comparison of the two species, the integral of AcOH (0.156) must be divided by three, giving 0.052. In the ^{19}F NMR spectrum, the signal of internal standard FBA falls at -116.2 ppm and accounts for one proton. The signal of F_4-BDC falls at -143.7 ppm and accounts for four protons. Thus, in order to have a quantitative comparison of the two species, the integral of F_4-BDC (2.188) must be divided by four, giving

0.547. This leads to calculate a F_4-BDC/AcOH ratio of 10.5. Assuming the general formula $Zr_6O_4(OH)_4(F_4\text{-BDC})_{6-x}(AcOH)_{2x}$, the following can be written:

$$\frac{6-x}{2x} = 10.5 \qquad \text{(equation } \mathbf{S3}\text{)}$$

Solving this equation, a value of 0.27 for *x* is determined, leading to the following proposed formula: $Zr_6O_4(OH)_4(F_4\text{-BDC})_{5.73}(AcOH)_{0.54}$, having FW of 2062 g mol^{-1}.

The absolute concentration of AcOH in solution is obtained by multiplying its normalised integral (0.052) times the concentration of FBA (0.11 M), obtaining 0.0057 M. The absolute amount (in mg) of AcOH in the MOF can be derived by multiplying the concentration times volume of the solution (1.0 mL) and the molecular weight of the acetate anion (59 g mol^{-1}), obtaining 0.34 mg. This leads to derive an experimental wt% of 1.59% for AcOH in the desolvated MOF. The absolute concentration of F_4-BDC in solution is obtained by multiplying its normalised integral (0.547) times the concentration of FBA (0.11 M), obtaining 0.0602 M. The absolute amount (in mg) of F_4-BDC in the MOF can be derived by multiplying the concentration times volume of the solution (1.0 mL) and the molecular weight of the F_4-BDC dianion (236 g mol^{-1}), obtaining 14.20 mg. This leads to derive an experimental wt% of 66.98% for F_4-BDC in the desolvated MOF. According to the proposed formula $Zr_6O_4(OH)_4(F_4\text{-BDC})_{5.73}(AcOH)_{0.54}$, the calculated wt% of AcOH is 1.54%, while that of F_4-BDC is 65.50%. The good agreement between experimental and calculated wt% suggests that the proposed formula is correct and that the analysed MOF does not contain impurities.

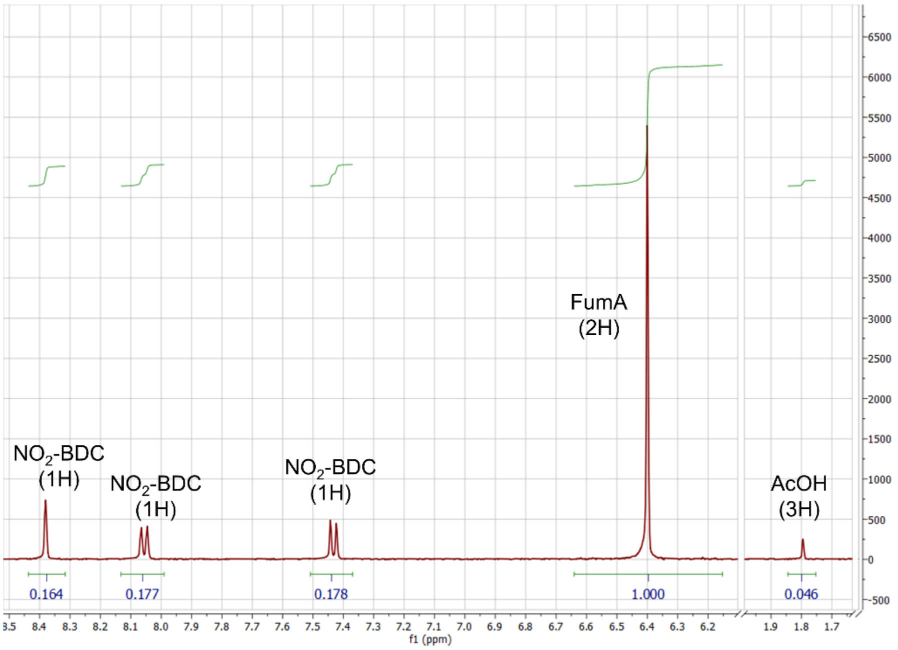

Figure S10. ^{1}H NMR spectrum of NO_2-UiO-66 0 μL water.

12.6 mg of desolvated MOF was digested. The signal of internal standard fumaric acid (FumA) falls at 6.40 ppm and accounts for two protons. The signals of NO_2-BDC fall at 8.38, 8.06 and 7.42 ppm and account for one proton each. The NO_2-BDC signal at higher chemical shift displays a slightly lower integral than the other signals, probably due to incomplete relaxation, therefore the integral of NO_2-BDC is taken as the mean between the integrals of the signals at 8.06 and 7.42 ppm, giving 0.178. The signal of AcOH falls at 1.80 ppm and accounts for three protons. Thus, in order to have a quantitative comparison of the three species, the integral of FumA (1.000) must be divided by two, giving 0.500, whereas the integral of AcOH (0.046) must be divided by three, giving 0.015. This leads to calculate a NO_2-BDC/AcOH ratio of 11.5. Assuming the general formula $Zr_6O_4(OH)_4(NO_2\text{-BDC})_{6-x}(AcOH)_{2x}$, the following can be written:

$$\frac{6-x}{2x} = 11.5 \qquad \text{(equation } \mathbf{S4}\text{)}$$

Solving this equation, a value of 0.25 for x is determined, leading to the following proposed formula: $Zr_6O_4(OH)_4(NO_2\text{-BDC})_{5.75}(AcOH)_{0.50}$, having FW of 1909 g mol^{-1}.

The absolute concentration of NO_2-BDC in solution is the ratio between the average integral (0.178) and 0.500, multiplied by the concentration of FumA (0.10 M), obtaining 0.0434 M. The absolute amount (in mg) of NO_2-BDC in the MOF can be derived by multiplying the

concentration times the volume of the solution (1.0 mL) and the molecular weight of the corresponding dianion (209 g mol^{-1}), obtaining 7.40 mg. This leads to derive an experimental wt% of 58.72% for NO_2-BDC in the desolvated MOF. The absolute concentration of AcOH in solution is the ratio between its normalised integral (0.015) and 0.500, multiplied by the concentration of FumA (0.10 M), obtaining 0.0031 M. The absolute amount (in mg) of AcOH in the MOF can be derived by multiplying the concentration times volume of the solution (1.0 mL) and the molecular weight of the acetate anion (59 g mol^{-1}), obtaining 0.18 mg. This leads to derive an experimental wt% of 1.44% for AcOH in the desolvated MOF. According to the proposed formula $Zr_6O_4(OH)_4(NO_2\text{-BDC})_{5.75}(AcOH)_{0.50}$, the calculated wt% of NO_2-BDC is 62.94%, while that of AcOH is 1.55%. The fact that slightly less NO_2-BDC and AcOH than expected are found suggests that there could be a small amount of impurity, probably of inorganic nature, in the product.

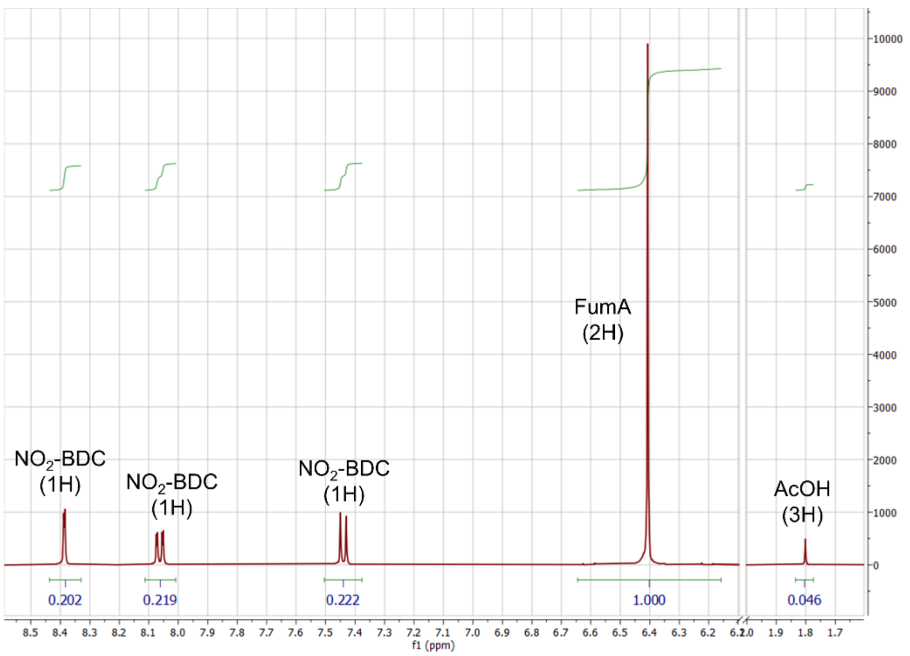

Figure S11. ^{1}H NMR spectrum of NO_2-UiO-66 25 μL water.

15.8 mg of desolvated MOF was digested. The signal of internal standard fumaric acid (FumA) falls at 6.40 ppm and accounts for two protons. The signals of NO_2-BDC fall at 8.38, 8.06 and 7.42 ppm and account for one proton each. The NO_2-BDC signal at higher chemical shift displays a slightly lower integral than the other signals, probably due to incomplete relaxation, therefore the integral of NO_2-BDC is taken as the mean between the integrals of the signals at 8.06 and 7.42 ppm, giving 0.221. The signal of AcOH falls at 1.80 ppm and accounts for three protons. Thus, in order to have a quantitative comparison of the three species, the integral of FumA (1.000) must be divided by two, giving 0.500, whereas the integral of AcOH (0.046) must be divided by three, giving 0.015. This leads to calculate a NO_2-BDC/AcOH ratio of 14.7. Assuming the general formula $Zr_6O_4(OH)_4(NO_2\text{-BDC})_{6-x}(AcOH)_{2x}$, the following can be written:

$$\frac{6-x}{2x} = 14.7 \qquad \text{(equation } \mathbf{S5}\text{)}$$

Solving this equation, a value of 0.20 for *x* is determined, leading to the following proposed formula: $Zr_6O_4(OH)_4(NO_2\text{-BDC})_{5.80}(AcOH)_{0.40}$, having FW of 1914 g mol^{-1}.

The absolute concentration of NO_2-BDC in solution is the ratio between the average integral (0.221) and 0.500, multiplied by the concentration of FumA (0.10 M), obtaining 0.0442 M.

The absolute amount (in mg) of NO_2-BDC in the MOF can be derived by multiplying the concentration times the volume of the solution (1.0 mL) and the molecular weight of the corresponding dianion (209 g mol^{-1}), obtaining 9.24 mg. This leads to derive an experimental wt% of 58.47% for NO_2-BDC in the desolvated MOF. The absolute concentration of AcOH in solution is the ratio between its normalised integral (0.015) and 0.500, multiplied by the concentration of FumA (0.10 M), obtaining 0.0031 M. The absolute amount (in mg) of AcOH in the MOF can be derived by multiplying the concentration times volume of the solution (1.0 mL) and the molecular weight of the acetate anion (59 g mol^{-1}), obtaining 0.18 mg. This leads to derive an experimental wt% of 1.15% for AcOH in the desolvated MOF. According to the proposed formula $Zr_6O_4(OH)_4(NO_2\text{-BDC})_{5.80}(AcOH)_{0.40}$, the calculated wt% of NO_2-BDC is 63.34%, while that of AcOH is 1.23%. The fact that slightly less NO_2-BDC and AcOH than expected are found suggests that there could be a small amount of impurity, probably of inorganic nature, in the product.

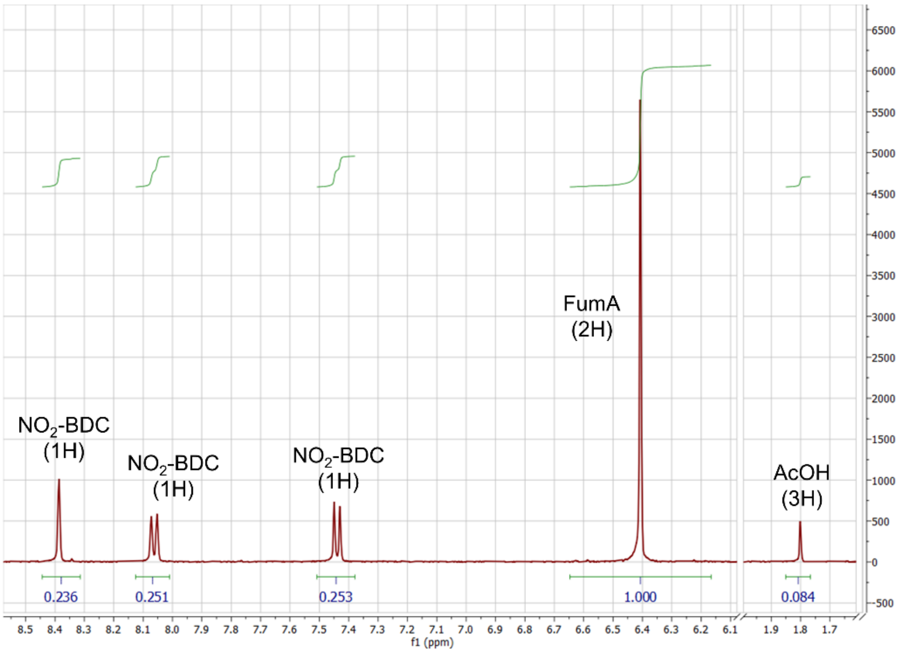

Figure S12. ^{1}H NMR spectrum of NO_2-UiO-66 50 µL water.

19.3 mg of desolvated MOF was digested. The signal of internal standard fumaric acid (FumA) falls at 6.40 ppm and accounts for two protons. The signals of NO_2-BDC fall at 8.38, 8.06 and 7.42 ppm and account for one proton each. The NO_2-BDC signal at higher chemical shift displays a slightly lower integral than the other signals, probably due to incomplete relaxation, therefore the integral of NO_2-BDC is taken as the mean between the integrals of the signals at 8.06 and 7.42 ppm, giving 0.252. The signal of AcOH falls at 1.80 ppm and accounts for three protons. Thus, in order to have a quantitative comparison of the three species, the integral of FumA (1.000) must be divided by two, giving 0.500, whereas the integral of AcOH (0.084) must be divided by three, giving 0.028. This leads to calculate a NO_2-BDC/AcOH ratio of 9.0. Assuming the general formula $Zr_6O_4(OH)_4(NO_2\text{-BDC})_{6-x}(AcOH)_{2x}$, the following can be written:

$$\frac{6-x}{2x} = 9.0 \qquad \text{(equation } \mathbf{S6}\text{)}$$

Solving this equation, a value of 0.32 for *x* is determined, leading to the following proposed formula: $Zr_6O_4(OH)_4(NO_2\text{-BDC})_{5.68}(AcOH)_{0.64}$, having FW of 1903 g mol^{-1}.

The absolute concentration of NO_2-BDC in solution is the ratio between the average integral (0.252) and 0.500, multiplied by the concentration of FumA (0.10 M), obtaining 0.0504 M.

The absolute amount (in mg) of NO_2-BDC in the MOF can be derived by multiplying the concentration times the volume of the solution (1.0 mL) and the molecular weight of the corresponding dianion (209 g mol^{-1}), obtaining 10.53 mg. This leads to derive an experimental wt% of 54.56% for NO_2-BDC in the desolvated MOF. The absolute concentration of AcOH in solution is the ratio between its normalised integral (0.028) and 0.500, multiplied by the concentration of FumA (0.10 M), obtaining 0.0056 M. The absolute amount (in mg) of AcOH in the MOF can be derived by multiplying the concentration times volume of the solution (1.0 mL) and the molecular weight of the acetate anion (59 g mol^{-1}), obtaining 0.33 mg. This leads to derive an experimental wt% of 1.71% for AcOH in the desolvated MOF. According to the proposed formula $Zr_6O_4(OH)_4(NO_2\text{-BDC})_{5.68}(AcOH)_{0.64}$, the calculated wt% of NO_2-BDC is 62.39%, while that of AcOH is 1.98%. The fact that slightly less NO_2-BDC and AcOH than expected are found suggests that there could be a small amount of impurity, probably of inorganic nature, in the product. The larger discrepancy found in this sample, compared to those prepared in the presence of less water, suggests that a larger amount of water leads to formation of more impurities.

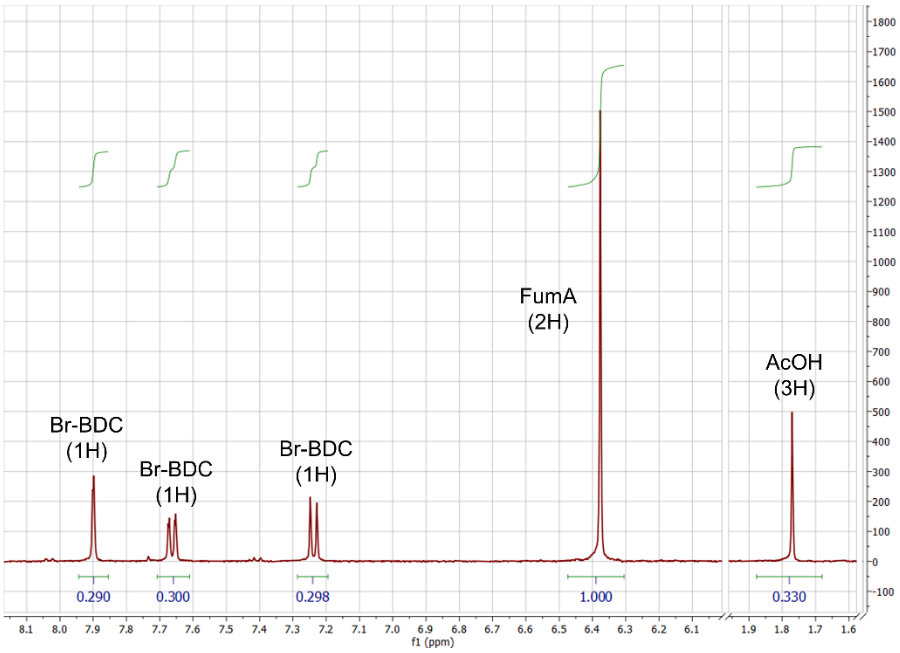

Figure S13. ^{1}H NMR spectrum of Br-UiO-66 100 μL water.

27.6 mg of desolvated MOF was digested. The signal of internal standard fumaric acid (FumA) falls at 6.37 ppm and accounts for two protons. The signals of Br-BDC fall at 7.90, 7.66 and 7.24 ppm and account for one proton each. The Br-BDC signal at higher chemical shift displays a slightly lower integral than the other signals, probably due to incomplete relaxation, therefore the integral of Br-BDC is taken as the mean between the integrals of the signals at 7.66 and 7.24 ppm, giving 0.299. The signal of AcOH falls at 1.78 ppm and accounts for three protons. Thus, in order to have a quantitative comparison of the three species, the integral of FumA (1.000) must be divided by two, giving 0.500, whereas the integral of AcOH (0.330) must be divided by three, giving 0.110. This leads to calculate a Br-BDC/AcOH ratio of 2.7. Assuming the general formula $Zr_6O_4(OH)_4(Br\text{-}BDC)_{6-x}(AcOH)_{2x}$, the following can be written:

$$\frac{6-x}{2x} = 2.7 \qquad \text{(equation } \mathbf{S7}\text{)}$$

Solving this equation, a value of 0.94 for *x* is determined, leading to the following proposed formula: $Zr_6O_4(OH)_4(Br\text{-}BDC)_{5.06}(AcOH)_{1.88}$, having FW of 2019 g mol^{-1}.

The absolute concentration of Br-BDC in solution is the ratio between the average integral (0.299) and 0.500, multiplied by the concentration of FumA (0.10 M), obtaining 0.0598 M.

The absolute amount (in mg) of Br-BDC in the MOF can be derived by multiplying the concentration times the volume of the solution (1.0 mL) and the molecular weight of the corresponding dianion (243 g mol^{-1}), obtaining 14.53 mg. This leads to derive an experimental wt% of 52.65% for Br-BDC in the desolvated MOF. The absolute concentration of AcOH in solution is the ratio between its normalised integral (0.110) and 0.500, multiplied by the concentration of FumA (0.10 M), obtaining 0.0220 M. The absolute amount (in mg) of AcOH in the MOF can be derived by multiplying the concentration times volume of the solution (1.0 mL) and the molecular weight of the acetate anion (59 g mol^{-1}), obtaining 1.30 mg. This leads to derive an experimental wt% of 4.70% for AcOH in the desolvated MOF. According to the proposed formula $Zr_6O_4(OH)_4(Br\text{-}BDC)_{5.06}(AcOH)_{1.88}$, the calculated wt% of NO_2-BDC is 52.39%, while that of AcOH is 5.50%. The good agreement between experimental and calculated wt% suggests that the proposed formula is correct and that the analysed MOF does not contain impurities.

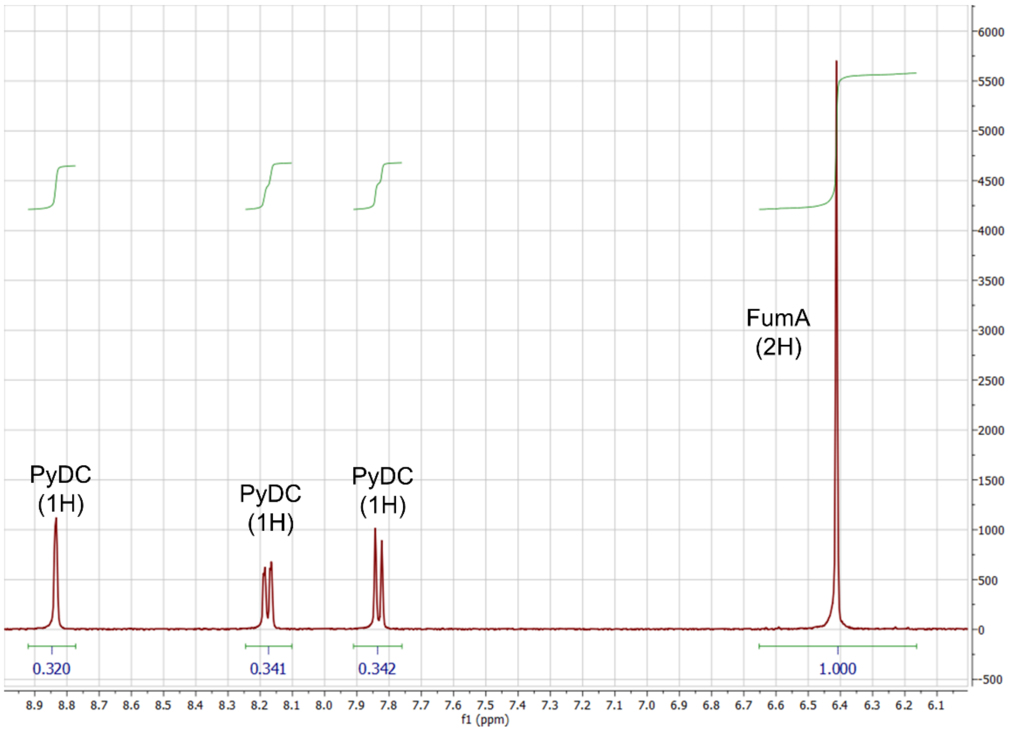

Figure S14. ^{1}H NMR spectrum of Py-UiO-66 1h.

23.5 mg of desolvated MOF was digested. The signal of internal standard fumaric acid (FumA) falls at 6.40 ppm and accounts for two protons. The signals of PyDC fall at 8.83, 8.18 and 7.82 ppm and account for one proton each. The PyDC signal at higher chemical shift displays a slightly lower integral than the other signals, probably due to incomplete relaxation, therefore the integral of PyDC is taken as the mean between the integrals of the signals at 8.18 and 7.82 ppm, giving 0.342. Thus, in order to have a quantitative comparison of the two species, the integral of FumA (1.000) must be divided by two, giving 0.500.

The absolute concentration of PyDC in solution is the ratio between the average integral (0.342) and 0.500, multiplied by the concentration of FumA (0.10 M), obtaining 0.0684 M. The absolute amount (in mg) of PyDC in the MOF can be derived by multiplying the concentration times the volume of the solution (1.0 mL) and the molecular weight of the corresponding dianion (165 g mol^{-1}), obtaining 11.29 mg. This leads to derive an experimental wt% of 48.03% for PyDC in the desolvated MOF. If the formula $Zr_6O_4(OH)_4(PyDC)_{6.00}$ is proposed (FW = 1668 g mol^{-1}), the calculated wt% of PyDC is 59.35%, a value much in excess of the experimental one.

Given the use of concentrated HNO_3 in this synthesis, it is reasonable to expect that the MOF could contain the PyDC linker with the pyridine nitrogen in protonated form and nitrate

as the counterion. Chromatographic analysis of the digested MOF revealed that it contained 1.67 mmol g^{-1} of NO_3^- (10.29 wt%). Thus, a PyDC/NO_3^- ratio of 1.75 can be derived. Assuming the formula $Zr_6O_4(OH)_4(PyDC)_{6-x}(HPyDC)_x(NO_3)_x$, the following can be written:

$$\frac{6}{x} = 1.75 \qquad \text{(equation } \mathbf{S8}\text{)}$$

Solving this simple equation, a value of 3.43 for *x* is determined, leading to the following formula: $Zr_6O_4(OH)_4(PyDC)_{2.57}(HPyDC)_{3.43}(NO_3)_{3.43}$, having FW of 1884 g mol^{-1}. According to this formula, the calculated wt% of PyDC is 52.55%, while that of NO_3^- is 11.29%. Thus, the proposed formula overestimates the wt% of both PyDC and NO_3^-. This could be due either to the presence of some impurity or to the presence of defects, compensated in this case by the hydroxide/water couple often reported in the literature. In the latter case, the formula $Zr_6O_4(OH)_4(PyDC)_{2.21}(HPyDC)_{2.95}(NO_3)_{2.95}(OH^-/H_2O)_{1.68}$ (FW = 1774 g mol^{-1}) can be proposed, which leads to calculated wt% of PyDC (48.00%) and NO_3^- (10.31%) in excellent agreement with the experimental ones.

7- PXRD PATTERNS X-UiO-66 1h, 2h

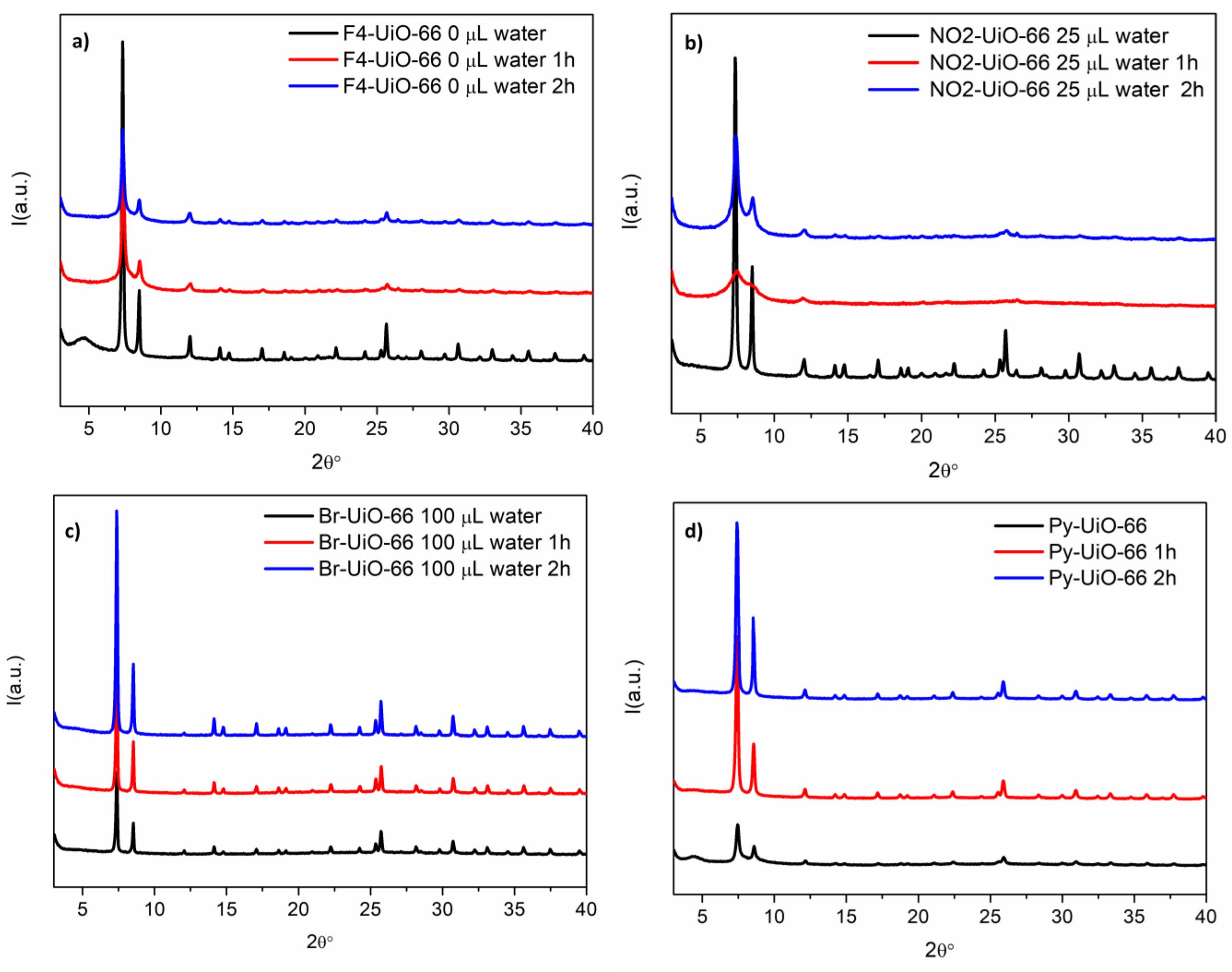

Figure S15. PXRD patterns of a) F_4-UiO-66 0 µL water synthesized in 1h and 2h, b) NO_2-UiO-66 25 µL water synthesized in 1h and 2h, c) Br-UiO-66 100 µL water synthesized in 1h and 2h, and d) Py-UiO-66 synthesized in 1h and 2h.

8- SCALE UP SYNTHESES

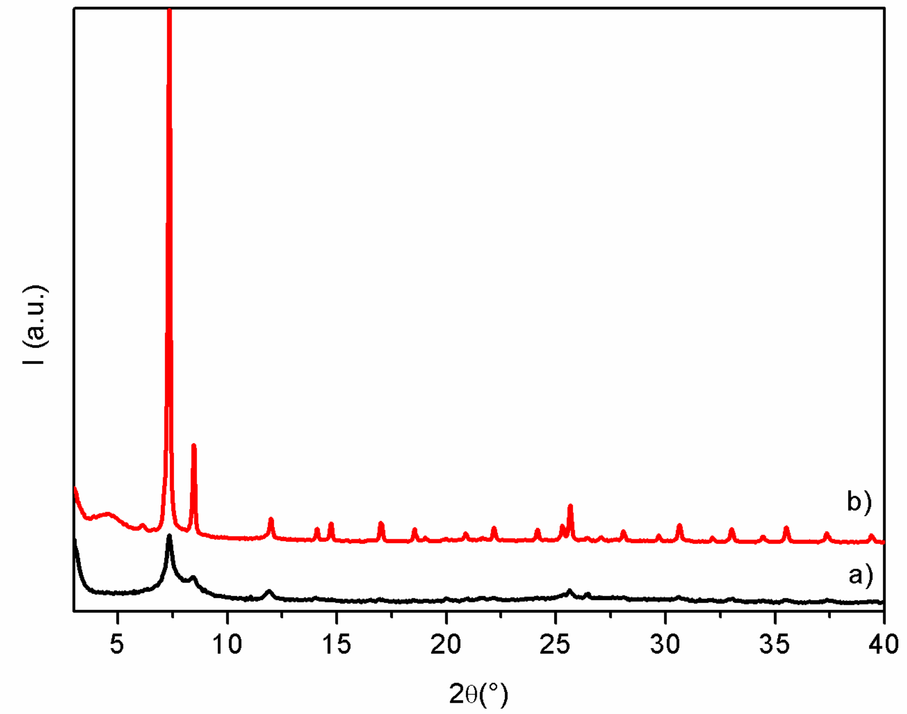

Figure S16. F_4-UiO-66 4x scaled up obtained with ball mill at 30 Hz after 1 h (a) and the same synthesis kept sealed for 24 h (b).

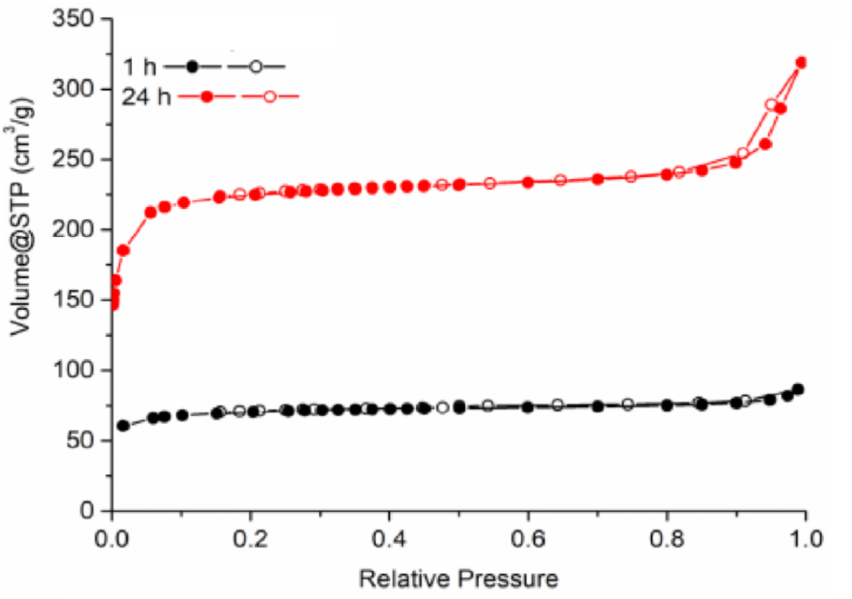

Figure S17. N_2 adsorption isotherms of F_4-UiO-66 obtained with ball mill at 30 Hz for 1h (black line) and the same synthesis kept sealed into the reactor for 24h (red line).

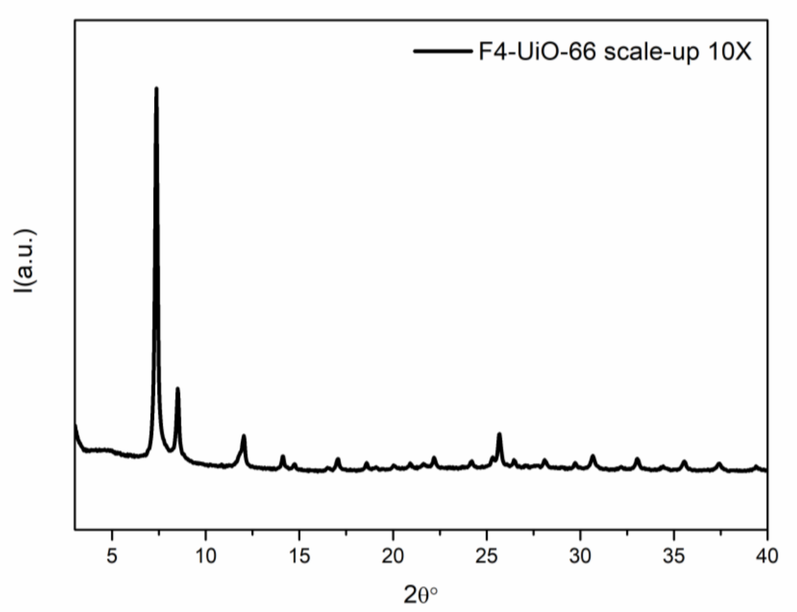

Figure S18. PXRD pattern for the scale-up synthesis of F4-UiO-66
